# The Bunyamwera orthobunyavirus Gc glycoprotein head and stalk drives an infectious virion assembly pathway specific for the insect host

**DOI:** 10.1371/journal.ppat.1014374

**Published:** 2026-07-07

**Authors:** Amelia B. Shaw, Hiu Nam Tse, Molly V. Durawa, Owen Byford, Hayley M. Pearson, Kenneth A. Stapleford, Juan Fontana, John N. Barr

**Affiliations:** 1 School of Molecular and Cellular Biology, Faculty of Biological Sciences, University of Leeds, Leeds, United Kingdom; 2 Astbury Centre for Structural Molecular Biology, University of Leeds, Leeds, United Kingdom; 3 Department of Microbiology, New York University Grossman School of Medicine, New York, United States of America; 4 Instituto Biofisika, CSIC-UPV/EHU, Barrio Sarriena s/n, Leioa, Bizkaia, Spain; University of New Mexico School of Medicine, UNITED STATES OF AMERICA

## Abstract

The *Orthobunyavirus* genus of arthropod-borne segmented RNA viruses comprises important pathogens including the human-infecting Oropouche virus and ruminant-infecting Schmallenberg virus (SBV). The prototypical Bunyamwera orthobunyavirus (BUNV) possesses envelope-embedded glycoprotein Gn-Gc tripodal spikes, of which the ectodomains mediate virus entry, while endodomains interact with nucleoprotein (NP) enwrapped genome segments driving virion assembly. Interestingly, BUNV Gc head/stalk domains are redundant for virus growth in mammalian cells, consistent with isolations of SBV from ruminants bearing head/stalk deletions. However, these domains appear strictly maintained in orthobunyavirus isolations from arthropods in nature. To investigate the molecular mechanism that underlines this discrepancy, we compared the multiplication characteristics of wildtype BUNV (BUNV-WT) with a Gc head/stalk deleted BUNV (BUNV-∆7). In mammalian cells BUNV-WT and BUNV-∆7 grew to equivalent titres, whereas in insect cells BUNV-∆7 titres were 1000-fold lower and strikingly produced no virions following blood meal infection of *Aedes* mosquitoes. To understand this insect-specific restriction in virion production, we showed the intracellular abundance of BUNV-WT and BUNV-∆7 Gc and NP components were equivalent, suggesting the deletion impacted post-translational stages of the infection cycle. To explore this, we investigated Gc and ∆7-Gc interactions during BUNV-WT and BUNV-∆7 infections of both insect and mammalian cells by co-immunoprecipitation and multiplex mass spectrometry, revealing ∆7-Gc exhibited markedly reduced NP interactions in insect cells, potentially indicating reduced segment interactions during assembly. We hypothesize that the Gc head/stalk performs an insect cell-specific role in segment recruitment during virion formation, and that maintenance in nature of full-length Gc is due to this essential role in the insect host.

## Author summary

Orthobunyaviruses (OBVs) are arthropod-borne viruses that are maintained in nature through a dual host animal/insect transmission cycle. Arboviral OBV infections are persistent whereas in humans and ruminants OBV infection can be associated with severe disease, including congenital malformations and abortions. OBV Gn and Gc glycoproteins form tripod spikes that protrude from the viral surface, which mediate entry and genome recruitment during assembly. Curiously, in nature, OBVs bearing large deletions within the tripod-forming Gc head/stalk domains have been isolated from diseased ruminants, yet the head/stalk domains appear to be maintained within insects. To investigate this discrepancy, we compared the multiplication of Bunyamwera orthobunyavirus (BUNV-WT) with a head/stalk-deleted variant (BUNV-∆7). In mammalian cells both viruses reached similar titres, but strikingly BUNV-∆7 failed to produce virions in either insect cells or *Aedes* mosquitoes. We showed this was because the BUNV-∆7 head/stalk-deleted Gc was deficient in interactions with genome components. We suggest that Gc drives species-specific interactions with genome segments during virion assembly, and hypothesize that Gc head/stalk domains are conserved in nature due to their essential role in the insect host.

## Introduction

The *Orthobunyavirus* genus within the *Bunyaviricetes* class is a large group of arthropod-borne segmented negative-sense RNA viruses that inflict a significant disease burden in mammals. Bunyamwera virus (BUNV) is the prototype of the orthobunyavirus (OBV) group and causes febrile illness in humans, although the BUNV reassortant Ngari virus has been associated with fatal outbreaks of haemorrhagic fevers [1]. The OBV group also includes Oropouche virus (OROV), capable of placental infection with subsequent foetal injury including microcephaly [2], and La Crosse virus, responsible for widespread outbreaks of encephalitis [3]. Several OBVs also have a significant impact on animal health, including Aino virus (AINOV), Akabane virus (AKAV) and Schmallenberg virus (SBV), which are capable of vertical transmission from mother to neonate, resulting in severe congenital malformations and foetal abortion [4]. All OBVs are maintained in nature by a vertebrate-arthropod transmission cycle, in which persistently infected arthropods act as vectors, transmitting the virus to vertebrates during a blood meal [5].

The OBV genome comprises three segments; the S segment encodes the nucleocapsid protein (NP) and the non-structural S protein (NSs) [5]. The M segment encodes a glycoprotein precursor (GPC) which is co-translationally cleaved to yield the glycoproteins Gn and Gc, and the non-structural M protein (NSm) [5]. Finally, the L segment encodes the large protein (L) which represents the virus-encoded component of the RNA-dependent RNA polymerase (RdRp) [5]. All three RNA segments associate with the RdRp and are protectively encapsidated with NP forming helical and pseudo-circular structures known as ribonucleoprotein (RNP) complexes [6].

OBVs, like other bunyaviruses, enter mammalian cells using their glycoprotein spikes, which protrude from the viral envelope. The spikes interact with various cellular receptors, such as lipoprotein-related protein 1 (LRP-1) recently identified as the receptor for OROV [7]. Following internalization into endosomes, the spikes also direct fusion of the viral and host membranes resulting in release of RNP segments into the cytosol where transcription and subsequently replication occur [8–10]. In mammalian cells, assembly of new OBV particles occurs within a re-organised Golgi, where newly made RNPs associate with the cytoplasmic tails of Gn/Gc glycoproteins [11, 12]. A similar mechanism has been observed for other bunyaviruses [13, 14]. Nascent OBV virions form and accumulate within the Golgi lumen to subsequently bud into exocytic vesicles and mature during trafficking to the plasma membrane for release [15–17]. The OBV infection cycle within insect cells is less well understood, although BUNV does not cause a similar ultrastructural reorganisation or swelling of the Golgi, with newly generated BUNV virions proposed to be released from insect cells immediately after formation without accumulation, facilitating long-term persistent infections [18, 19].

The OBV Gc protein is sub-divided into three structural domains: the head, stalk and floor. The ultrastructural organisation of the BUNV glycoproteins on the viral surface has been characterised, displaying a tripodal organisation. Here, three Gc head domains form the apex of the tripod, connected to the base of the tripod through the stalk domain [8,20,21]. The tripod base is composed of three Gc floor domains, which contain the class II fusion domain, alongside three copies of Gn, forming a trimer of heterodimers, which stabilises the tripodal structure and shields the fusion domain [8]. Both Gn and Gc possess C-terminal cytoplasmic tails that protrude inside the virion, and in the absence of a canonical viral matrix protein, have been proposed to interact with the RNPs, driving virion assembly and budding [11].

The Gc head region is highly variable and several viable OBV variants with mutations and deletions in the Gc head region have been identified. Headless variants were first described in 1981 during passage of Maguari virus (MAGV) in mammalian cells in the presence of the mutagen 5-fluorouracil (S1A Fig) [22, 23]. Mutations in the SBV Gc head domain were also identified following serial passage in mammalian IFN-incompetent cell lines [24]. For BUNV, a systematic deletion analysis of Gc revealed the head/stalk domains to be largely dispensable for multiplication in mammalian cells [25] with one recombinant variant, termed delta-7 (rBUNV-∆7), exhibiting similar growth kinetics to wild-type BUNV despite removal of the entire head domain and most of the stalk region (S1B Fig) [25].

Interestingly, viruses bearing extensive Gc mutations have also been isolated in nature. For SBV, isolates extracted from ruminants revealed a mutational hotspot termed the hypervariable region (HVR) in the Gc head domain (S1C Fig) [26] and later studies identified multiple SBV mutants bearing deletions of up to 200 amino acids, with some encompassing portions of both Gc head and stalk domains [26–28]. However, no isolations of Gc-deleted OBVs from arthropods have been reported, suggesting the functional requirements of Gc may be different across the two hosts that make up the OBV sylvatic multiplication cycle.

Here, we wanted to further investigate the role of the OBV Gc head and stalk domains, to better understand why they are apparently dispensable for multiplication in mammalian cells, yet are maintained in nature. Using rBUNV, we compared the growth of both rBUNV-WT and rBUNV-∆7 in mammalian versus arthropod-derived cells. While structural proteins Gc and NP from both viruses accumulated with comparable abundance in all cell lines, in insect cells, we observed redistribution of ∆7-Gc to the plasma membrane alongside a dramatic drop in released rBUNV-∆7 titres. These observations suggested rBUNV-∆7 was deficient in virion production. To investigate the basis of this deficit we used co-immunoprecipitation paired with tandem mass tag (TMT) mass spectrometry to examine Gc interactions within infected cells. This revealed a significant reduction in the ability of the head/stalk truncated Gc of rBUNV-∆7 to interact with NP in insect cells, which was not seen in mammalian cells. This suggests Gc potentially plays a role in RNP recruitment and its deletion prevents formation of new virions within the insect host. These findings provide a plausible explanation for why Gc-truncated OBVs can be isolated from mammalian hosts, but not from arthropods.

## Results

### rBUNV-Δ7 produces infectious virus in mammalian cells but not in insect cells

To better understand the role of the Gc head/stalk domain in OBV growth, we compared the replication characteristics of wildtype BUNV and the variant rBUNV-Δ7 missing the Gc head/stalk region. To achieve this, we rescued recombinant wildtype BUNV (rBUNV-WT) and the variant rBUNV-Δ7, as previously described [25] (S2A Fig), with successful rescue confirmed by western blot analysis of NP expression in both transfected and infected cell lysates (S2B Fig).

**Fig 1:**
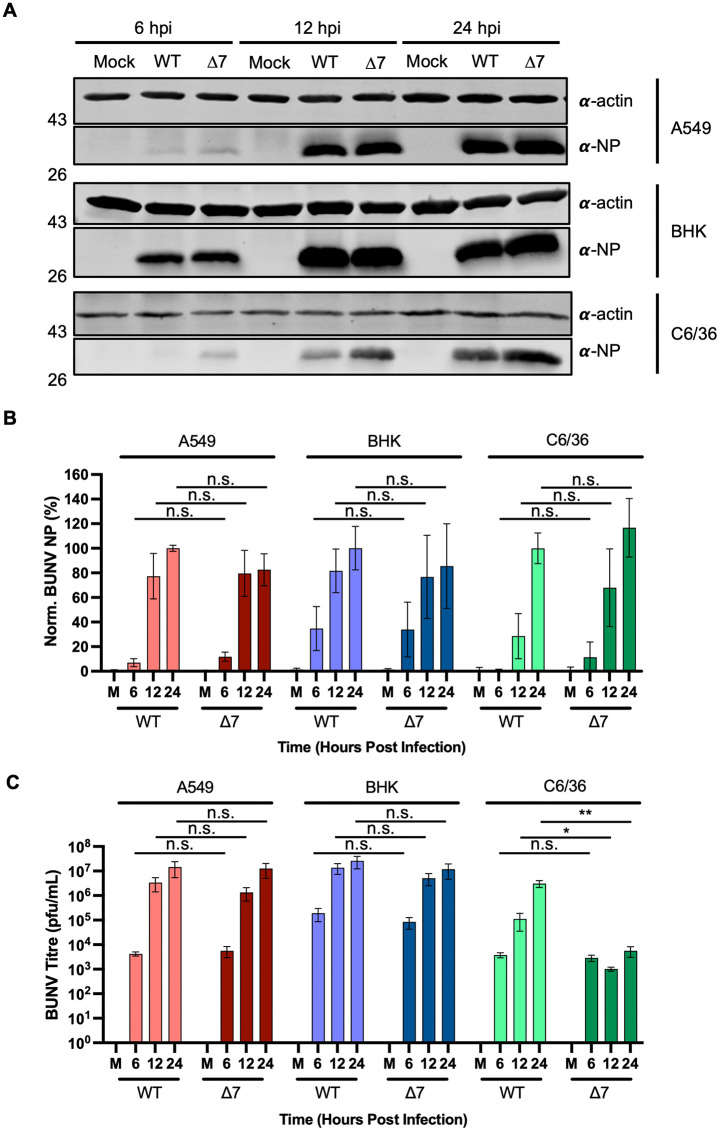
Comparison of single cycle growth kinetics between wildtype BUNV and ∆7 BUNV in multiple cell lines. A549 cells (mammalian), BHK cells (mammalian) and C6/36 cells (insect) were either mock-infected or infected with rBUNV-WT (WT) or mutant rBUNV-∆7 (∆7) at an MOI of 5 and supernatants and cell lysates were collected at either 6, 12 or 24 hours post infection (n = 3). **(A)** Cell lysates were subject to western blot analysis and probed for expression of NP and actin, as a loading control. **(B)** Densitometry analysis performed on (A) as expression of NP relative to actin. The expression of BUNV NP was normalised to average of WT at 24 hours post infection for each cell line. Results were analysed by Student’s t-test (unpaired) whereby n.s. = p > 0.05, comparing WT to ∆7 at each timepoint. **(C)** Titration of the supernatants was performed by plaque assay. Log_10_-transformed results were analysed by Student’s t-test (unpaired) whereby n.s. = p > 0.05, * = p < 0.05; ** = p < 0.01; *** = p < 0.001, comparing WT to ∆7 at each timepoint.

To reflect the two-host enzootic multiplication cycle of OBVs, we compared rBUNV-WT and rBUNV-Δ7 activities in mammalian-derived A549 and BHK cells as well as mosquito-derived C6/36 cells. To assess their multiplication characteristics, we performed a time-course experiment in which A549, BHK and C6/36 cells were infected with either rBUNV-WT or rBUNV-Δ7 at an MOI of 5, in an attempt to infect every cell simultaneously and focus on analysing a single infectious cycle. At 6-, 12- and 24-hours post infection (hpi), cellular lysates and supernatants were harvested (Fig 1). Lysates were probed for expression of NP using western blot analysis (Fig 1A) with subsequent densitometry (Fig 1B) revealing similar NP expression from both rBUNV-WT or rBUNV-Δ7 at all time points measured in A549 and BHK cells. In C6/36 cells, rBUNV-Δ7 NP appeared slightly earlier (Fig 1A–1B), but this was not statistically significant, and overall NP expression of the two viruses at 24 hpi was indistinguishable. This suggested rBUNV-WT and rBUNV-Δ7 were able to enter and perform gene expression across all three cell lines in an equivalent manner.

Next, we assessed the titre of infectious rBUNV-WT or rBUNV-Δ7 released from the three cell lines (Fig 1C) in harvested supernatants. The same three timepoints (6, 12 and 24 hpi) as for lysate harvesting were used; 6 hpi reflecting an early timepoint at which NP expression is low in A549 and C6/36 cells and no new viruses have been generated [25,29]. Similarly, 12 hpi has been shown to reflect a timepoint when NP is expressed in all three cell lines and at least one round of infection has been completed [25,29]. Finally, 24 hpi reflecting a timepoint at which multiple rounds of infection have occurred, infecting cells that were not initially infected. Supernatant titre analysis by plaque assay showed release of infectious rBUNV-WT and rBUNV-Δ7 from mammalian A549 and BHK cells similarly increased over all three timepoints, reaching approximate titres of 10^7^ pfu/mL at 24 hpi. Interestingly, while this was comparable to the release of infectious rBUNV-WT from C6/36 cells, the titre of infectious rBUNV-Δ7 from C6/36 cell supernatants was reduced by around 1000-fold and in fact did not increase beyond the 6 hpi titre suggesting no new viruses were released.

To assess the behaviour of rBUNV-∆7 following multiple rounds of replication, we also infected A549 or C6/36 cells with rBUNV-WT or rBUNV-∆7 at a lower MOI of 0.01. As for MOI 5, titration analysis (S3 Fig) revealed no significant difference in the titres of rBUNV-WT and rBUNV-∆7 released from A549 cells at either 24 hpi or 48 hpi, both reaching 10^8^ pfu/mL by 48 hpi. In contrast, in C6/36 cells, supernatant titres of rBUNV-∆7 were 5-logs lower than that of rBUNV-WT at both 24 hpi and 48 hpi (S3 Fig), reflecting the severe deficiency in virion production seen at MOI 5. Taken together, these results show that removal of head/stalk domain severely disrupts the production of progeny infectious BUNV from insect cells, but not in mammalian cells.

### rBUNV-Δ7 fails to establish infection in Aedes mosquitoes

**Fig 2:**
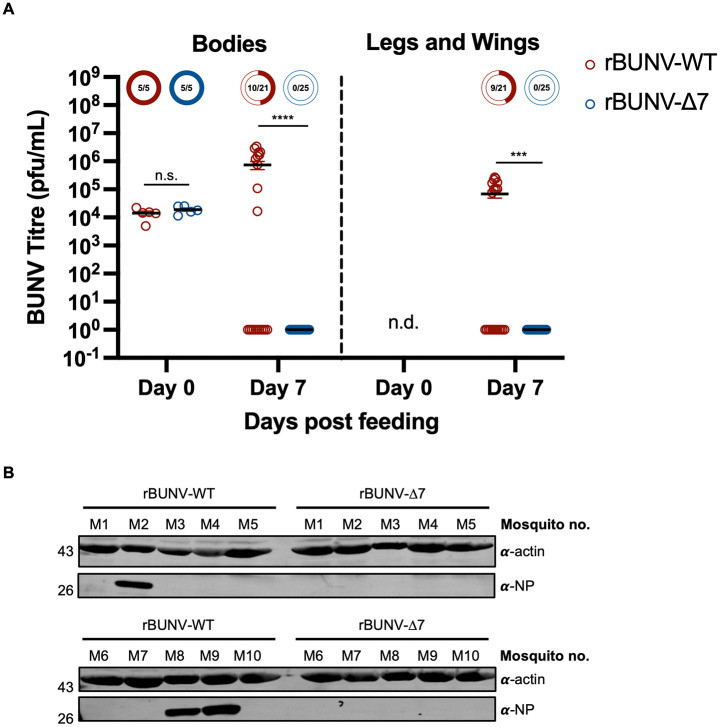
Comparison of infection of *Aedes* mosquitoes by wildtype BUNV and ∆7 BUNV. *Aedes* mosquitoes were fed a blood meal containing 1x10^7^ pfu/mL of rBUNV-WT or rBUNV-∆7. **(A)** Bodies or legs and wings were harvested either immediately (Day 0) or 7 days later (Day 7) and titre was calculated by plaque assay. Titres are displayed as pfu/mL and individual samples have been shown (n = 5 for both viruses at Day 0; n = 21 for rBUNV-WT and n = 25 for rBUNV-∆7 at Day 7). The bar represents the average value, with standard error of the mean. Titres were analysed by Mann Whitney test (unpaired) whereby n.s. = p > 0.05, *** = p < 0.001, **** = p < 0.0001, comparing WT to ∆7. **(B)** Mosquito lysates (10 mosquitoes for each virus) were subject to western blot analysis and probed for expression of NP and actin, as a loading control.

To assess whether the block in rBUNV-∆7 virion generation was specific to insect cells in culture, we sought to test the multiplication characteristics of rBUNV-∆7 in female *Aedes aegypti* mosquitoes. Briefly, over 20 mosquitoes were fed blood-meals containing either rBUNV-WT or rBUNV-∆7 at 1 × 10^7^ pfu/mL, with day 0 samples collected immediately after feeding to confirm successful ingestion (Fig 2A; Day 0). At 7 days post infection (dpi), mosquitoes were collected and titres were assessed (Fig 2A; Day 7) from the bodies, to determine infection progress, and from the legs and wings, to determine effective dissemination. Of all the mosquitoes fed with a blood-meal containing rBUNV-WT, 10/21 mosquitoes were successfully infected with rBUNV-WT and in 9 of those, BUNV had successfully disseminated to the legs and wings. Of the mosquitoes fed with a blood-meal containing rBUNV-∆7, 0/25 mosquitoes were infected with rBUNV-∆7 and there was no detectable virus in the legs and wings. This was corroborated by western blot analysis of mosquitoes fed with either rBUNV-WT (10 mosquitoes) or rBUNV-∆7 (10 mosquitoes), which showed 3/10 mosquitoes infected with rBUNV-WT expressed BUNV NP, whereas there was no NP expression in any of the mosquitoes infected with rBUNV-∆7 (Fig 2B). This showed that despite ingestion of rBUNV-∆7 into the mosquitoes, no subsequent virus amplification occurred, suggesting that the Gc head is necessary for successful viral multiplication within mosquitoes, corroborating our insect cell culture data.

### rBUNV-Δ7 expresses abundant Gc glycoprotein in insect cells

The results of the previous section showed titres of rBUNV-∆7 failed to rise above inoculum levels in C6/36 cells, and one possible reason for this was the failure to express sufficient glycoproteins for virion production. To assess this, we generated infectious rBUNV-WT and rBUNV-∆7 variants expressing HA-tagged versions of their corresponding Gc proteins, thus allowing abundance of both Gc and NP to be assessed by western blot analysis. The HA tag was introduced into the head region of WT-Gc and the remaining stalk region of ∆7-Gc (S4A Fig) and successful rescue of corresponding viruses rBUNV-WT-Gc-HA and rBUNV-Δ7-Gc-HA was confirmed by detection of NP and Gc-HA by western blot analysis of both transfected and infected cell lysates (S4B Fig). Expression of WT- and ∆7-Gc-HA at their respective expected molecular weights (S4B Fig), suggested that Gc-HA was correctly expressed and processed from the GPC precursor, despite the deletion of the head/stalk domain.

**Fig 3:**
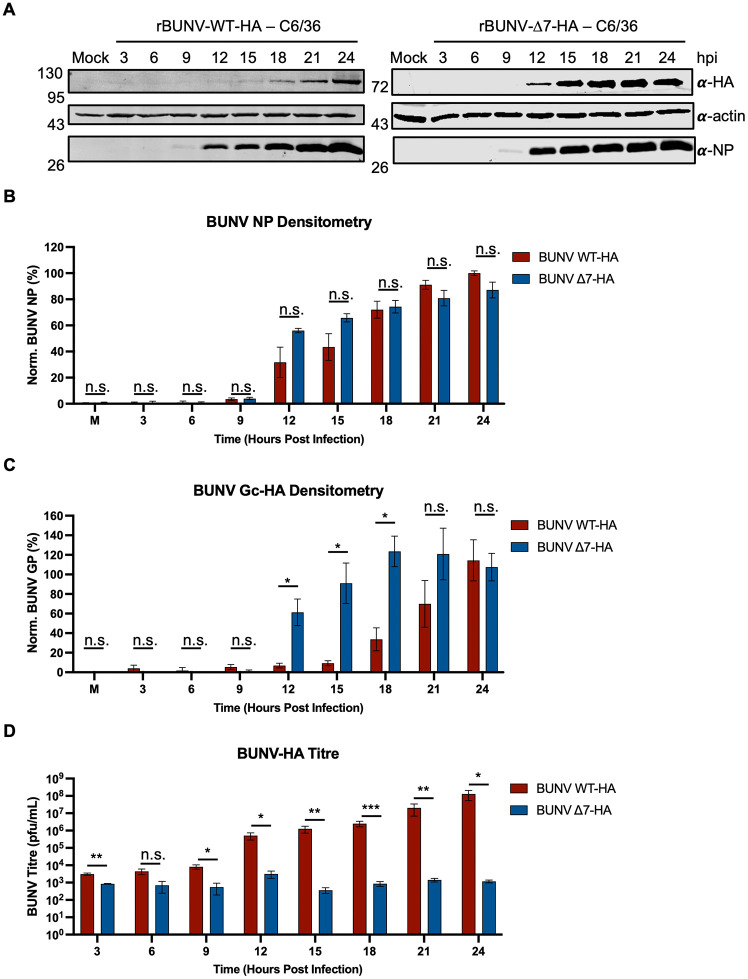
Comparison of growth kinetics and protein expression of wildtype rBUNV-HA and ∆7 rBUNV-HA in C6/36 cells. C6/36 cells were either mock-infected or infected with rBUNV-WT-Gc-HA (WT-HA) or mutant rBUNV-∆7-Gc-HA (∆7-HA) at an MOI of 5 and supernatants and cell lysates were collected at every 3 hours post infection (hpi) until 24 hpi (n = 3). **(A)** Cell lysates were subject to western blot analysis and probed for expression of NP, HA (for expression of Gc-HA) and actin, as a loading control. **(B)** Densitometry analysis performed on (A) as expression of NP relative to actin. The expression of BUNV NP was normalised to the average of rBUNV-WT-Gc-HA NP at 24 hours post infection. **(C)** Densitometry analysis performed on (A) as expression of Gc-HA relative to actin. The expression of BUNV Gc-HA was normalised to rBUNV-WT-Gc-HA Gc-HA at 24 hours post infection. **(D)** Titration of the supernatants was performed by plaque assay and titres were plotted on a logarithmic scale graph. All results were analysed by Student’s t-test (unpaired) whereby n.s. = p > 0.05, * = p < 0.05; ** = p < 0.01; *** = p < 0.001, comparing WT-HA to ∆7-HA at each timepoint.

The Gc-HA-tagged viruses were used to investigate expression of NP and Gc in insect cells. Here, C6/36 cells were infected with either rBUNV-WT-Gc-HA or rBUNV-Δ7-Gc-HA at an MOI of 5 and lysate and supernatant samples were collected every 3 hpi until 24 hpi (Fig 3). NP expression occurred slightly earlier in rBUNV-Δ7-Gc-HA-infected C6/36 cells when compared to rBUNV-WT-Gc-HA, but densitometry analysis showed any differences in NP expression levels were not statistically significant at any time point (Fig 3A–3B). Similarly, Gc-HA expression occurred earlier for rBUNV-Δ7-HA compared to rBUNV-WT-Gc-HA, although by 21 and 24 hpi, there was no significant difference in Gc expression levels. Titration of released C6/36 cell supernatants showed that multiplication of rBUNV-WT-Gc-HA was not affected by the addition of the HA tag, since titres were comparable to those of rBUNV-WT, and confirmed that the titres of rBUNV-∆7-Gc-HA were similarly significantly reduced in C6/36 cells (Fig 3D). Together, these data show that rBUNV-Δ7-HA and rBUNV-WT-Gc-HA express NP and Gc structural proteins with equivalent abundance, indicating that the defect in infectious rBUNV-Δ7 virion production occurs at a stage post protein production.

### rBUNV-∆7 does not accumulate within insect cells or synthesise non-infectious virus-like particles

**Fig 4:**
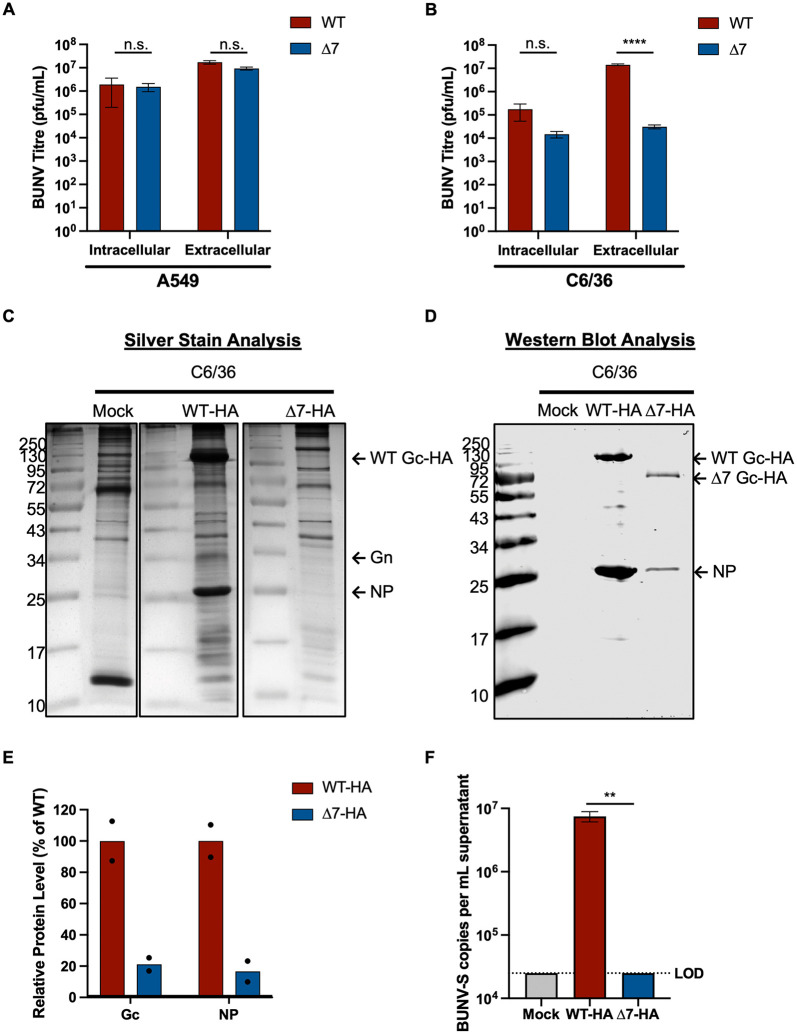
Comparison of intracellular and extracellular virus production of wildtype BUNV and ∆7 BUNV in A549 and C6/36 cells. A549 cells (A) or C6/36 cells (B) were infected with rBUNV-WT (WT; red) or mutant rBUNV-∆7 (∆7; blue) at an MOI of 5 and supernatants and cells were collected at 24 hours post infection (n = 3). Cells were then freeze-thawed three times in 1 × TNE buffer to disrupt the plasma membrane and release any assembled virions that were present within the cell (intracellular). Intracellular and extracellular (supernatant samples) were then titred by plaque assay. Log_10_-transformed samples were analysed by Student’s t-test (unpaired) whereby n.s. = p > 0.05, **** = p < 0.0001, comparing WT to ∆7 for each sample. **(C)** Extracellular supernatant from mock-infected C6/36 cells or C6/36 cells infected with rBUNV-WT-Gc-HA or rBUNV-∆7-Gc-HA was purified by ultracentrifugation (n = 2). The resuspended pellet was subject to silver stain analysis (C) and western blot analysis **(D)**, probing for expression of HA and NP. The protein ladder sizes are indicated (kDa) as well as the predicted bands for viral structural proteins WT-Gc, ∆7-Gc, Gn and NP. **(E)** Densitometry analysis performed on (D) as expression of ∆7-Gc-HA or NP relative to WT-Gc-HA or NP. The expression of the ∆7 proteins were normalised to the average expression of WT-Gc-HA or NP. **(F)** RNA was extracted from supernatant collected from mock-infected C6/36 cells or C6/36 cells infected with rBUNV-WT-Gc-HA or rBUNV-∆7-Gc-HA (n = 3). The extracted RNA was quantified by RT-qPCR and copy numbers of BUNV S genome per mL were calculated using a standard curve of known RNA concentrations ranging from 2.5 × 10^7^ to 2.5 × 10^1^ copies/mL. The dashed horizontal line represents the limit of detection (LOD), which was the lowest concentration of the standard curve (2.5 × 10^4^ copies/mL). Samples that failed to amplify within 25 PCR cycles were assigned the LOD value for statistical analysis. Log_10_-transformed samples were analysed by Welch’s unpaired t-test whereby ** = p < 0.01; comparing WT to ∆7.

The lack of infectious rBUNV-Δ7 released into C6/36 cell supernatants could have been due to a defect in virion assembly, or a defect in virion release. To distinguish between these possibilities, we next assessed whether infectious virions were trapped inside cells. A549 and C6/36 cells were infected with either rBUNV-WT or rBUNV-Δ7 at an MOI of 5, after which titres of both supernatant-associated extracellular virus and cell lysate-associated intracellular virus were assessed by plaque assay at 24 hpi. The titres of extracellular rBUNV-WT released from A549 and C6/36 cells were comparable (Fig 4A and 4B, extracellular), and consistent with previous 24 hpi supernatant titres, described above (Fig 1C), as was the statistically significant reduction of titre of extracellular infectious rBUNV-Δ7 in C6/36 cells (Fig 4B; extracellular). Critically, the titre of C6/36 intracellular infectious rBUNV-∆7 was statistically indistinguishable from that of rBUNV-WT (Fig 4B; intracellular), suggesting the dramatic reduction in rBUNV-∆7 supernatant titres was not due to an intracellular accumulation of assembled virions. In A549 cells, there was no significant difference between titres of intracellular or extracellular infectious rBUNV-∆7 and rBUNV-WT (Fig 4A).

As an orthogonal approach to detect accumulated intracellular virions, we directly observed sections of resin-embedded infected C6/36 cells by electron microscopy (S5 Fig). However, we were unable to detect any intracellular virions in C6/36 cells infected with either rBUNV-WT [19] (S5B Fig) or rBUNV-∆7 (S5C Fig).

Finally, to determine whether non-infectious rBUNV-∆7 virus-like particles (VLPs) were released, we sought to examine presence of VLPs in infected C6/36 cell supernatant. Supernatant was collected from C6/36 cells either mock-infected or infected with rBUNV-WT or rBUNV-∆7 and concentrated by ultracentrifugation through a sucrose cushion. The purified supernatant was examined by silver stain analysis (Fig 4C) and western blot analysis (Fig 4D). The silver stain analysis showed bands corresponding to the viral structural proteins Gc, Gn and NP for rBUNV-WT-Gc-HA, which were not clearly identifiable in the mock or rBUNV-∆7-Gc-HA purified supernatants. The western blot analysis clearly showed the presence of Gc-HA and NP in only rBUNV-WT-Gc-HA and rBUNV-∆7-Gc-HA purified supernatant and densitometry analysis (Fig 4E) revealed a five-fold reduction of both Gc and NP when comparing rBUNV-∆7-Gc-HA protein levels to those of rBUNV-WT-Gc-HA. Finally, RT-qPCR was also performed to identify relative levels of the S genome present in the supernatant (Fig 4F). This data showed that there was ~ 10^7^ BUNV S genome copies per mL supernatant from C6/36 cells infected with rBUNV-WT-Gc-HA. In comparison, BUNV S genomic RNA was below the limit of detection (2.5 × 10^4^ copies/mL) in supernatant from C6/36 cells either mock-infected or infected with rBUNV-∆7-Gc-HA. Taken together, this data is consistent with a lack of virus particle production from rBUNV-∆7-infected C6/36 cells.

Overall, our findings are consistent with a scenario in which lack of released infectious rBUNV-∆7 within C6/36 cells results from a deficiency in virion assembly.

### Differences between insect and mammalian cells do not explain reduced rBUNV-∆7 virus production from insect cells

To understand the molecular mechanism responsible for the lack of assembly and release of rBUNV-∆7 from insect cells, we considered differences between the mammalian and insect cell lines, including lower culture temperature and lower cholesterol levels in the cellular membrane. To investigate whether reducing the culture temperature affected growth of rBUNV-∆7, A549 and BHK cells were infected with either rBUNV-WT or rBUNV-∆7 at an MOI of 5 and incubated at 30 °C, instead of 37 °C. At 24 hpi, supernatant titres were measured, which showed both rBUNV-WT and rBUNV-∆7 reached equivalent high 10^6^ pfu/mL titres (S6A Fig). This suggests that reducing the culture temperature does not account for the reduction of rBUNV-∆7 titre in insect cells.

Unlike mammals, insects do not synthesise cholesterol, instead relying on dietary sources [30]. Given cholesterol abundance has been shown to regulate BUNV entry [31], we hypothesised that differences in cholesterol incorporation in the cellular membranes of the mammalian and insect cell lines could account for the block in infectious rBUNV-∆7 release. In an attempt to recover rBUNV-∆7 viability, we supplemented C6/36 insect cell cultures with cholesterol during infection using an established cholesterol supplementation protocol [32]. To do this, C6/36 cells were pre-treated with either 0.05 mg/mL or 0.1 mg/mL cholesterol, alongside methyl-β-cyclodextrin (MβCD; 85 µM) as a vehicle control, before infecting with rBUNV-WT or rBUNV-∆7 at MOI 5 and harvesting supernatant at 24 hpi (S6B Fig). Titration of the supernatants showed that supplementing C6/36 cells with cholesterol did not affect rBUNV-WT titre, as expected, nor did it recover rBUNV-∆7 titre. This suggests that the absence of cholesterol in insect cells is not the reason for the reduction in released rBUNV-∆7 titres.

### Loss of glycosylation and trimerisation sites does not account for the reduction in rBUNV-∆7 titres from insect cells

WT-Gc and ∆7-Gc sequences differ in their glycosylation potential due to the loss of residue N624 as a consequence of the head/stalk deletion. To investigate whether glycosylation differences could account for reduced rBUNV-∆7 supernatant titres, we removed this glycosylation site from rBUNV-WT by the substitution N624Q to generate mutant rBUNV-N624Q. We hypothesized that if this residue played a critical role in infectious rBUNV-∆7 production, then titres of rBUNV-N624Q from C6/36 cells would also be low. A549 cells or C6/36 cells were infected with rBUNV-WT, rBUNV-∆7 or rBUNV-N624Q at MOI of 1 (due to lower titre of rBUNV-N624Q) and incubated for 24 hours. Titration of the supernatant showed that rBUNV-WT and rBUNV-∆7 were again similar in A549 cells and the rBUNV-∆7 titre was reduced in C6/36 cells (S6C Fig). Interestingly, rBUNV-N624Q titre was reduced in both A549 and C6/36 cells, but not to the same extent as rBUNV-∆7 in C6/36 cells (S6C Fig). Whilst these results show that glycosylation plays a role in viability of BUNV, they also show that glycosylation does not fully explain the lack of assembled rBUNV-∆7 viruses in insect cells.

A final potential difference between rBUNV-WT and rBUNV-∆7 is the lack of Gc head, which mediates trimerisation of the tripod. To determine whether spike tripod formation is important for BUNV virion production in insect cells, we identified potential head trimerisation residues in Gc (S575, R583, Q589 and D592) using PDBePISA (Proteins, Interfaces, Structures and Assemblies) and substituted each for alanine in the rBUNV-WT context to generate infectious mutant rBUNV-∆tripod. As above, we infected A549 and C6/36 cells with rBUNV-∆tripod at an MOI of 1 (due to lower titre of rBUNV-∆tripod) and harvested supernatant at 24 hpi (S6C Fig). There was a slight reduction in rBUNV-∆tripod titre, in both cell lines, when compared to rBUNV-WT but again this was not as significant a reduction as rBUNV-∆7 in C6/36 cells, suggesting the alanine substitutions introduced in an attempt to disrupt Gc head trimerisation do not account for the reduced rBUNV-∆7 viability is reduced in C6/36 cells.

### ∆7-Gc is transported to the plasma membrane in insect cells

**Fig 5:**
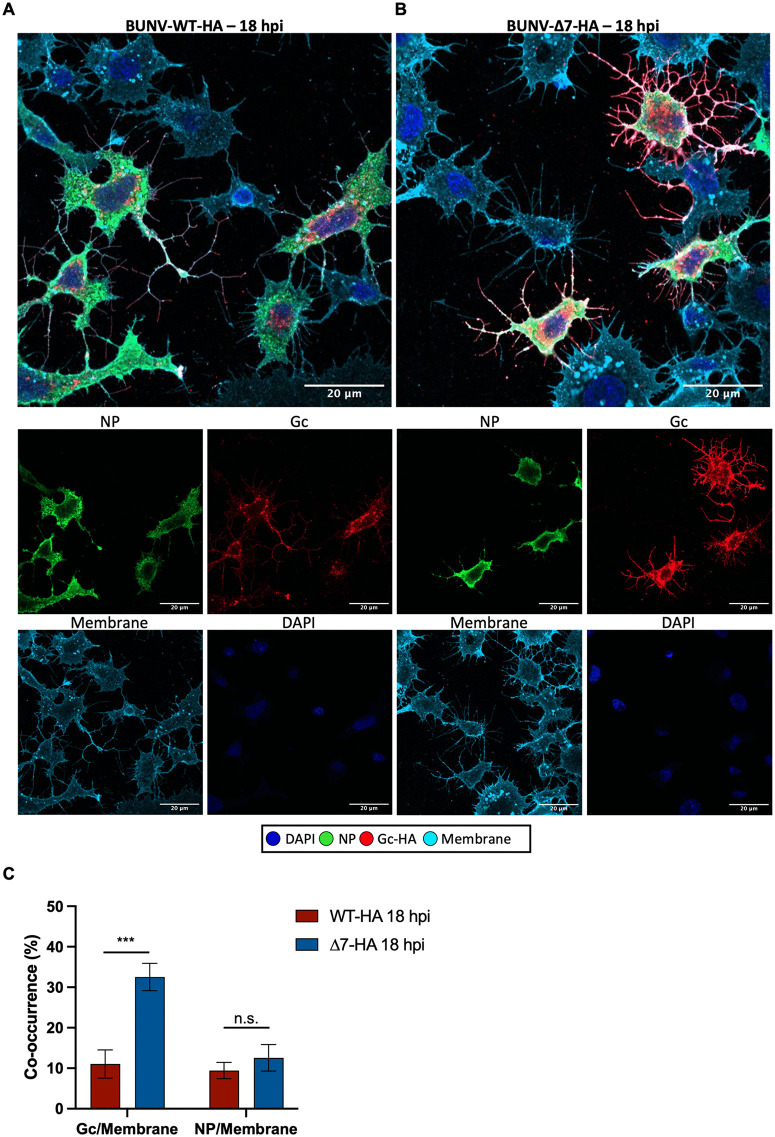
Confocal microscopy images of C6/36 cells infected with wildtype rBUNV-HA or ∆7 rBUNV-HA at 18 hours post infection. C6/36 cells were infected with rBUNV-WT-Gc-HA (A) or rBUNV-∆7-Gc-HA (B) at an MOI of 5 and fixed with formaldehyde at 18 hpi. The cells were then permeabilized, blocked, and stained for the nucleus (DAPI, blue), concanavalin A (membrane, cyan), BUNV NP (green), and BUNV Gc-HA (red) by indirect immunostaining. The cells were imaged on LSM990 confocal microscope at ×63 magnification. Scale bars representing 20 µm are shown. DAPI, 4,’6-diamidino-2-phenylindole. **(C)** Co-occurrence analysis was performed by analysing the proportion of Gc signal or NP signal that was present in the same area as a mask representing the plasma membrane of the cells. Approximately 20 cells were selected for each condition to be analysed. All results were analysed by Student’s t-test (unpaired) whereby n.s. = p > 0.05; ** = p < 0.01, comparing WT-HA to ∆7-HA for each protein.

To investigate whether Gc localisation is affected by deletion of the head/stalk region, C6/36 cells were infected with either rBUNV-WT-Gc-HA or rBUNV-Δ7-Gc-HA, fixed at 18 hpi and 24 hpi, stained for NP, Gc-HA and cellular membranes and imaged using confocal microscopy (Figs 5; 18 hpi and S7; 24 hpi). 18 hpi was chosen to reflect a time when WT-Gc expression was detectable but there was a significant difference between the levels of WT-Gc and ∆7-Gc expression, whereas 24 hpi was chosen to reflect a time when the difference between WT-Gc and ∆7-Gc abundance was not significant (Fig 3C). At 18 hpi and 24 hpi, NP distribution in rBUNV-WT-Gc-HA- and rBUNV-Δ7-Gc-HA-infected cells was indistinguishable, presenting as a diffuse cytoplasmic staining (Figs 5A and 5B, and S7A and S7B, green).

However, in contrast to NP, localization of WT-Gc-HA and ∆7-Gc-HA in virus infected cells exhibited clear differences. At 18 hpi, WT-Gc-HA expressed in rBUNV-WT-Gc-HA-infected C6/36 cells appeared to localise exclusively within dense punctate regions, likely Golgi, in agreement with previous work [19] (Fig 5A, red). However, distribution of ∆7-Gc-HA at 18 hpi showed strong staining at the plasma membrane (Fig 5B, red) in addition to the aforementioned cytoplasmic puncta. At 24 hpi, when overall Gc expression levels are similar for WT-Gc-HA and ∆7-Gc-HA, staining of the respective WT-Gc-HA and ∆7-Gc-HA spikes was predominantly within cytoplasmic puncta (S7 Fig, red) however, ∆7-Gc-HA was still abundantly detected at the plasma membrane, with a much weaker signal for WT-Gc-HA. Using the membrane stain, a mask was generated to delineate the plasma membrane and thus permit quantitative analysis of the redistribution of Gc to this site (Figs 5C and S7C). This showed, at both timepoints, that approximately 30% of the ∆7-Gc-HA signal co-occurred with the membrane mask. This was a statistically significant increase over the proportion of WT-Gc-HA signal co-occurred with the membrane mask, which was approximately 10%. In contrast to this, NP from both rBUNV-WT-Gc-HA-infected and rBUNV-∆7-Gc-HA-infected C6/36 cells showed low co-occurrence with the membrane mask, owing to the predominant cytoplasmic distribution, and there was no significant difference between WT and ∆7.

**Fig 6:**
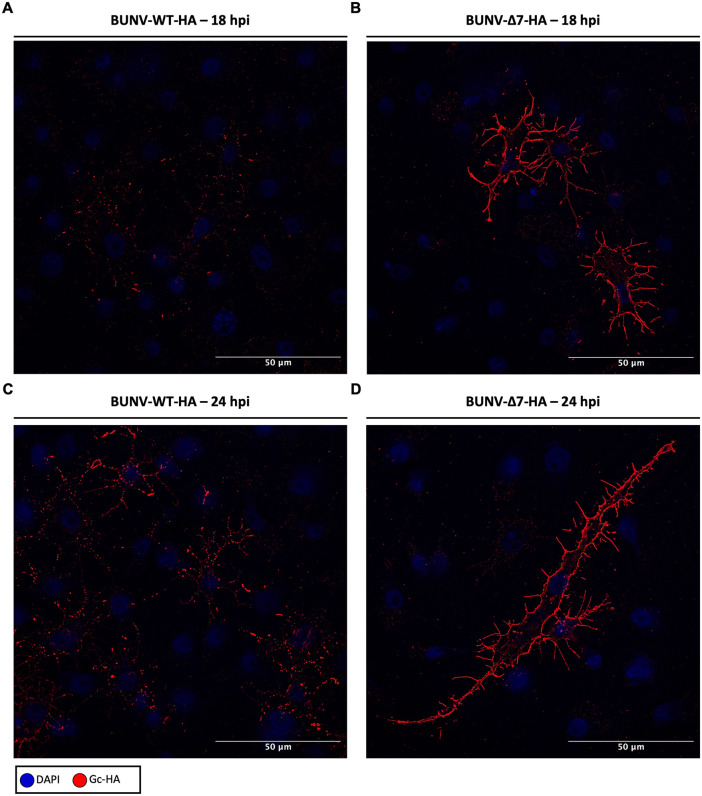
Widefield microscopy images of non-permeabilised C6/36 cells infected with wildtype rBUNV-HA or ∆7 rBUNV-HA. C6/36 cells were infected with rBUNV-WT-Gc-HA (A and C) or rBUNV-∆7-Gc-HA (B and D) at an MOI of 5 and fixed with formaldehyde at either 18 (A and B) or 24 (C and D) hpi. The cells were not permeabilized, but were blocked, and stained for the nucleus (DAPI, blue) and BUNV Gc-HA (red) by indirect immunostaining. The cells were imaged on an Olympus IX83 widefield microscope at ×100 magnification. Z stack images were collected, and deconvolution (with 3 iterations) was performed. The slice showing the strongest signal was chosen for the display image. Scale bars representing 50 µm are shown. DAPI, 4,’6-diamidino-2-phenylindole.

Finally, to test whether ∆7-Gc was indeed present at the surface of the plasma membrane, C6/36 cells were infected with either rBUNV-WT-Gc-HA or rBUNV-Δ7-Gc-HA and fixed at 18 hpi and 24 hpi. Then, without permeabilization, the cells were stained for surface expression of Gc-HA and imaged using widefield microscopy (Fig 6). At both 18 hpi and 24 hpi timepoints, Δ7-Gc-HA showed a strong localisation to the plasma membrane (Fig 6B and 6D), whereas WT-Gc-HA showed a more punctate localisation at the plasma membrane, potentially representing released virions (Fig 6A and 6C). Together, these observations suggest the cellular distribution of Δ7-Gc-HA and WT-Gc-HA is different in insect cells, potentially explaining the differences we observe in viral titres.

### The head/stalk Gc deletion decreases Gc-NP association in insect cells, potentially blocking RNP recruitment

**Fig 7:**
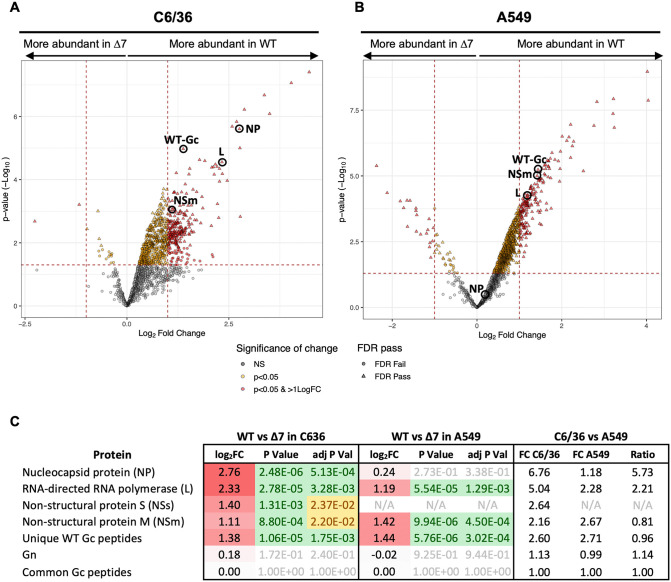
Mass spectrometry analysis of proteins identified from immunoprecipitation with BUNV Gc-HA. A549 cells or C6/36 cells were mock-infected or infected with rBUNV-WT-Gc-HA (WT-HA) or mutant rBUNV-∆7-Gc-HA (∆7-HA) at an MOI of 1 for 24 hours post infection. TMT-MS was then performed as described in methods, and normalised abundances were statistically analysed using the limma package. The volcano plots for the results from C6/36 cells (A) and A549 cells (B) were created by plotting the -log_10_ p-value of each protein against the log_2_ fold change. Proteins where p < 0.05 are highlighted in yellow, and proteins where p < 0.05 and log_2_FC is > 1 or <-1 are highlighted in red. **(C)** Log_2_FC, p-value and adjusted p-value (adj p-val) for each viral protein identified in both cell lines is shown. Log_2_FC has been colour-coded as a gradient whereby 2 = red and 0 = white. The p-values and adj p-values have been colour-coded whereby green indicates p < 0.01, yellow indicates p < 0.05 and white indicates p > 0.05. N/A means that no peptides were identified for that protein in that cell line.

The results described above showed that Δ7-Gc-HA and WT-Gc-HA exhibit differences in their sub-cellular distributions within infected cells, and we hypothesized this could affect interactions with other virion components required for the assembly of infectious virions. To test this, we used co-immunoprecipitation followed by mass spectrometry analysis to identify intracellular binding partners of Δ7-Gc-HA and WT-Gc-HA. Briefly, A549 or C6/36 cells were infected with rBUNV-WT-Gc-HA or rBUNV-Δ7-Gc-HA at an MOI of 1 (due to lower titres of rBUNV-WT-Gc-HA and rBUNV-Δ7-Gc-HA) and incubated for 24 hpi, when cell lysates were collected. The lysates were then incubated overnight with an anti-HA antibody, which was previously incubated with Protein G-coupled magnetic beads. After washing, the beads were collected and subjected to mass spectrometry analysis (Fig 7) or western blot analysis (S8 Fig), to confirm similar protein abundances in the samples. Western blot (S8A–S8B Fig) and densitometry analysis (S8C Fig) suggested that, despite equivalent expression of Gc-HA and NP in the lysate samples (S8A Fig), ∆7-Gc-HA was less efficient than WT-Gc-HA at co-precipitating NP in C6/36 cells but not in A549 cells (S8B Fig). Densitometry analysis confirmed there was a statistically significant reduction in NP precipitated by ∆7-Gc-HA in C6/36 cells so we sought to investigate this further.

Tandem mass-tag liquid chromatography mass spectrometry (TMT-MS) was performed on bead fractions precipitated from three biological repeats of either mock-, rBUNV-WT-Gc-HA- or rBUNV-∆7-Gc-HA-infected A549 cells or C6/36 cells, allowing relative quantification of the interacting proteins of WT-Gc-HA and ∆7-Gc-HA. The log_2_-transformed abundances were normalised to the abundance of shared Gc sequences present in each condition (Fig 7C; Common Gc peptides), to compensate for any potential differences in the immunoprecipitation of WT-Gc-HA and ∆7-Gc-HA. Log_2_ fold changes (log_2_FC) were calculated for each protein, resulting in positive values (indicating WT-Gc-HA precipitated more protein than ∆7-Gc-HA), values close to 0 (indicating equal precipitation between WT-Gc-HA and ∆7-Gc-HA) or negative values (indicating ∆7-Gc-HA precipitated more protein than WT-Gc-HA) (Fig 7C). Volcano plots were then generated for each cell line to reflect log_2_FC changes and significance (p-value) (Fig 7A–7B; S1 Data).

The analysis revealed a significantly higher abundance of NP peptides precipitated from rBUNV-WT-Gc-HA-infected C6/36 cells compared to rBUNV-∆7-Gc-HA-infected C6/36 cells (log_2_FC = 2.76, adj p-value = 5.13E-04). Interestingly, the log_2_FC of NP peptides precipitated from rBUNV-WT-Gc-HA- compared to rBUNV-∆7-Gc-HA-infected A549 cells was 0.24 (adj p-value = 3.38E-0.1), suggesting there was no significant difference between the precipitation of NP by WT-Gc-HA or ∆7-Gc-HA in A549 cells. Furthermore, this was not due to a lack of NP precipitation by Gc-HA because there was a similar significant enrichment of NP present in both WT and ∆7 A549 infections over mock (WT; log2FC = 3.93, p-value = 3.20E-03, ∆7; log2FC = 4.04, adj p-value = 4.83E-03) (S1 Data). There were also similar changes in the other bunyaviral proteins (L, NSm and NSs) in which the WT-Gc-HA was able to precipitate higher abundances than ∆7-Gc-HA, generally to a greater extent in C6/36 cells than A549 cells (Fig 7C). In contrast to this, Gn had close to 0 log_2_FC values in both cell lines, suggesting Gn was equally precipitated by WT- and ∆7-Gc-HA. Finally, the Gc sequences unique to WT-Gc-HA were similarly enriched in both cell lines (C6/36; log_2_FC = 1.38, adj p-value = 1.75E-03, A549; log_2_FC = 1.44, adj p-value = 3.02E-04; WT Gc peptides in Fig 7C), which is to be expected due to the lack of some of these peptides in ∆7-Gc-HA. Together, this suggests that the ability of ∆7-Gc-HA to interact with NP is dramatically reduced in C6/36 cells, but not in A549 cells, consistent with the species-specific deficiency in insect host virion production.

## Discussion

OBV glycoproteins are multifunctional, necessary for viral entry, assembly, exit and evasion of the host immune system. Remarkably, OBVs bearing large deletions within the Gc glycoprotein head and stalk domains can be isolated from infected ruminants, whereas these domains appear to be strictly conserved in OBV isolations from arthropods.

To better understand this discrepancy, we investigated the behaviour of a BUNV variant lacking the majority of the Gc head and stalk domains (rBUNV-∆7; [25]) in cultured mammalian and insect cells, as well as live mosquitoes. We showed that rBUNV-∆7 was capable of entering both mammalian and insect cells, and performing broadly equivalent gene expression to that of rBUNV-WT. However, whereas rBUNV-∆7 generated equivalent titres to BUNV-WT in mammalian cells, infectious virion production was greatly impaired in cultured C6/36 cells. Furthermore, there were no infectious rBUNV-∆7 virions recovered from *Aedes aegypti* mosquitoes, despite ingestion of the virus. This suggests that rBUNV-∆7 is similarly deficient in virion production following initial infection of cells within the mosquito, although further investigation into how rBUNV-∆7 behaves during the initial stages of infection of the mosquito would be required to rule out entry defects or innate immune response in context of the whole organism. Furthermore, it would be interesting to determine whether rBUNV-∆7 is capable of entry, replication and virion production within the mammalian host, both in immunocompetent and immunodeficient animals. Despite the need for further investigations into the whole organism, our results do indicate that the OBV Gc head and stalk domains play a species-specific role in generation of infectious virions.

To understand the molecular basis for why ∆7-Gc caused a species-specific block in infectious virion production, we tested host cell-based differences such as cholesterol content and optimal growth temperature, alongside viral Gc-specific differences including differential glycosylation potential and ability to form the characteristic OBV tripodal spike. While none of these variables impaired infectious virion production, we showed that deletion of the head/stalk influenced Gc cellular localisation in insect cells, with significantly increased abundance of ∆7-Gc at the plasma membrane compared to WT-Gc.

To investigate whether this differential localization of ∆7-Gc and WT-Gc had an impact on the protein-protein interactions of Gc, we co-immunoprecipitated both ∆7-Gc and WT-Gc from insect cell lysates, revealing a significant reduction in the interaction between ∆7-Gc and RNP components NP and L, compared to WT-Gc. These reduced interactions suggest a role of Gc in RNP recruitment into newly generated virions in insect cells. Crucially, immunoprecipitation of ∆7-Gc and WT-Gc from mammalian cell lysates did not result in differential pull-down of RNP components, a finding also consistent with the similar titres of released rBUNV-WT and rBUNV-∆7 viruses in these cells. Taken together, these findings indicate that the presence of the Gc head and stalk alters Gc protein interactions, which results in the different infection outcomes in mammalian and insect cells.

One possible reason for the species-specific infection outcome could be the abundance of a cell factor specific to either insect or mammalian cells that facilitates the Gc-RNP interaction. Related to this, the TMT-MS analysis identified several insect proteins that had significantly reduced interactions with ∆7-Gc compared to WT-Gc. These included components of the COPI coatomer complex and various mitochondrial proteins, associated with vesicular transport or potential virus factory function, respectively [15,33,34]. While these associations are provocative, their role in the post-translation stages of the infection cycle remains to be determined. Interestingly, several other proteins with reduced ∆7-Gc interactions were categorised as uncharacterised in terms of function, with no mammalian homologues, which is suggestive of insect cell-specific pathways that could potentially play important roles in virion assembly (S1 Data). Further investigation of these hits could offer insight into what happens in the later stages of infection in insect cells. From our results, we were unable to conclude which specific stage of the assembly pathway was blocked by deletion of the Gc head and stalk domains, although our results confirm that no new infectious virions are produced, there is not an accumulation of intracellular virions, and it does not appear that virions are accumulated at the plasma membrane, unable to be released. Therefore, this would suggest that ∆7-Gc virions in insect cells are unable to assemble due to the reduced Gc-NP interaction, but further investigation would be required to fully elucidate what is happening.

Alternatively, the Gc head and stalk could dictate species-specific infection outcomes through differences in Gc expression kinetics or trafficking, where loss of the head/stalk could affect the availability of spike proteins to interact with RNPs at destined assembly sites. Specifically, a reduction of ∆7-Gc-NP interaction could be the result of the redistribution of ∆7-Gc to the plasma membrane in insect cells and this would warrant further investigation. A similar scenario was proposed for Crimean Congo haemorrhagic fever virus (CCHFV), for which deletion of GP38 affected Gc maturation and reduced Gc localisation to the Golgi, and deletion of NSm increased the rate of Gc trafficking and resulted in reduced N-glycosylation [35].

It is interesting to speculate how removal of the Gc head might affect interaction with NP for the late stages of infection, particularly as the Gc head and tail locate within the Golgi lumen, while NP is present at the cytosol. This being the case, it is highly unlikely that the ∆7-Gc deletion prevents a direct interaction between NP and its head/stalk. The Gc-NP interaction is thought to be driven by the cytoplasmic tails of Gn and Gc, fulfilling the role of a canonical viral matrix protein and mediating recruitment of all viral components necessary for virion generation [11]. Our TMT-MS analysis showed that the interaction between ∆7-Gc and Gn was not significantly different to that of WT-Gc, which confirms removal of the Gc head does not affect the heterodimeric complex. However, whether trimerisation of the floor domain, or the Gc and Gn cytoplasmic tails, is disrupted is not known. In an attempt to decipher this, we generated a rBUNV-∆tripod mutant to mimic the lack of trimerisation driven by the Gc head. This produced high titres from C6/36 cells, unlike rBUNV-∆7, suggesting lack of tripod formation (i.e., trimerisation of the head domain) is not required for virion production. There is also the potential that the complex glycosylation seen in mammalian cells is required to direct proper folding and multimerization of Gc, even in its truncated version, whereas the simpler glycosylation in insect cells might not be sufficient to drive correct folding. However, ∆7-Gc is missing only one glycosylation site compared to WT-Gc, and mutant virus rBUNV-N624Q showed that removal of this site did not result in the same low titres as rBUNV-∆7 from insect cells. The deleted sequence corresponds to nearly 350 amino acids and therefore it is possible that there are other unknown modification sites or signals, potentially insect-cell-specific, present in this region.

The observation that the Gc head and stalk domains are dispensable for entry in both mammalian and insect cells suggests that either removal of the Gc head does not disrupt receptor binding/attachment, or that exposure of the fusion domain offers a receptor-independent entry route, at least *in vitro*. The redundancy of these domains in mammalian cells is consistent with the low sequence conservation of the OBV Gc N-terminus [22–27], with this antigenic drift likely driven by the need to escape neutralizing antibodies that target these regions [21,32,36]. Taken together, the role of the OBV Gc head and stalk regions are likely to offer a prominent target to the mammalian adaptive immune response, misdirecting antibody-mediated neutralization from the functionally-critical residues involved in membrane fusion that are located in the Gc C-terminus, and whose sequence is more rigid due to a high functional constraint [37]. However, the results presented here suggest the Gc head and stalk also provide further critical roles in the virus multiplication cycle aside from a neutralizing antibody decoy.

Our results offer a plausible explanation for why naturally occurring SBV mutants bearing deletions of the Gc head domain can be detected in the ruminant foetus, but not in infected viraemic mothers, nor in insects [26–28,38]. We propose that Gc head/stalk deletion mutants arise due to an inherent mutational hotspot in the Gc N-terminus, sometimes leading to genetic drift, but also sometimes leading to large scale sequence deletion of the entire head, often along with deletions in the stalk [26,27]. In immunocompetent ruminant mothers, such deletions would likely be selected against due to exposure of functionally critical C-terminal Gc sites, and subsequent antibody-mediated neutralization [21,28,37,38]. In contrast, due to the lack of a robust adaptive immune response, such head/stalk deleted mutants are capable of rapid amplification in the early-stage foetus, resulting in the devastating pathogenicity associated with neonatal OBV infection [26,28,39]. On the other hand, while the strong selection pressure for maintenance of the Gc head/stalk in the infected ruminant may be a contributing factor, the lack of an adaptive immune response in insects would not provide any pressure for the Gc head/stalk maintenance in mosquitoes. Therefore, we propose that maintenance of the OBV Gc head/stalk in insects primarily results from the species-specific assembly deficiency described here. Interestingly, Gc may not be the only OBV protein able to cause a species-specific block in virus multiplication, as recent reports describe an SBV mutant bearing a single NP mutation as responsible for a lack of virus multiplication within both cultured *Culicoides* midge cells [40] as well as live midges [41]. Taken together, these findings highlight major knowledge gaps exist in understanding the complex interplay of determinants of virus fitness in the dual host OBV multiplication cycle.

## Materials and methods

### Cell lines

BHK-21 cells, BSR-T7 cells and A549 cells were acquired from ATCC and maintained in high-glucose Dulbecco’s modified Eagle medium (DMEM; Sigma-Aldrich) supplemented with 10% heat-inactivated foetal bovine serum (FBS), 100 µg of streptomycin/mL and 100 U of penicillin/mL, and incubated in a humidified incubator at 37 °C with 5% CO_2_. BSR-T7 cells constitutively expressing T7 RNA polymerase (T7P) [42] were also additionally supplemented with G418 (500 µg/mL) every other passage to maintain the T7P plasmid. C6/36 insect cells were gifted by Andrew Tuplin (University of Leeds) and were maintained at 28 °C in Leibovitz’s L-15 media (Thermo Fisher Scientific) which was supplemented with 10% Tryptose Phosphate Broth (Thermo Fisher Scientific) and 100 µg of streptomycin/mL and 100 U of penicillin/mL.

### Plasmids

Plasmids encoding the full-length cDNAs that represent the intact S (pT7riboBUNS(+); Genbank accession number: NC_001927.1), M (pT7riboBUNM(+); Genbank accession number: NC_001926.1) and L (pT7riboBUNL(+); Genbank accession number: NC_001925.1) segments of BUNV were gifted by Alain Kohl (University of Glasgow) [43]. Site-directed mutagenesis was used to delete amino acids D480 to N826 from pT7riboBUNM(+) to generate pT7riboBUNMΔ7(+), as previously described [25].

Plasmids pT7riboBUNM-HA(+) and pT7riboBUNMΔ7-HA(+) were synthesised through PCR insertion of the HA tag (YPYDVPDYA), flanked by flexible 4xG/S sequences, into the Gc region (inserted between amino acids T502 and D503 in pT7riboBUNM-HA(+) or between amino acids V482 and Y483 in pT7riboBUNMΔ7-HA(+) (equivalent residues in WT are V829 and Y830). Plasmid pT7riboBUNM-trimer(+) was synthesised through PCR alanine substitution of residues S575, R583, Q589, D592. Plasmid pT7riboBUNM- N624Q (+) was synthesised by PCR substitution of residue N624 to glutamine. All primers sequences are available upon request.

### Generation of recombinant BUNV from cDNA

BSR-T7 cells were seeded in 6-well plates at a density of 2 × 10^5^ cells/well. Following overnight incubation, the cells were transfected with 1 µg of pT7riboBUNS(+), 1 µg of pT7riboBUNM(+), 1 µg of pT7riboBUNL(+) and 0.3 µg of pUC57-T7 in 200 µL OptiMEM media, followed by 2.5 µL/µg TransIT-LT1 transfection reagent (Mirus). For recovery of the Δ7 variant, pT7riboBUNM(+) was replaced with pT7riboBUNMΔ7(+). Tagged or mutant versions of viruses involved replacing the appropriate plasmids by their tagged or mutated counterparts. Control transfections were also performed, in which pT7riboBUNL(+) was excluded. At 4-hour post transfection (hpt), the media was removed and replaced with 2 mL of DMEM supplemented with 2.5% FBS. At 120 hpt, the supernatants were collected, clarified and used to infect BHK-21 cells, seeded at a cell density of 5 × 10^5^ cells in T25 flasks the previous day. 48–72 hours post infection (hpi), upon evidence of cytopathic effect, supernatant was collected, centrifuged at 4000 *× g* for 15 mins, filtered through a 0.45 µm filter and aliquoted into cryo-vials for storage at -80 °C. To prepare bulk stocks of virus, T175 flasks were seeded with 1.5 × 10^7^ BHK-21 cells, incubated for 24 h and then infected with BUNV (or the BUNV variants) at an MOI of 0.01. At 48 hpi, supernatant was clarified and filtered as described above.

### Viral protein expression and virus release in mammalian and insect cells

A549 (1 × 10^5^ cells/well), BHK-21 (1 × 10^5^ cells/well) and C6/36 (4 × 10^5^ cells/well) were seeded in 12-well plates and incubated at either 37 °C (mammalian) or 28 °C (insect). The cells were then infected with rBUNV-WT or rBUNV-Δ7 at an MOI of 5 and incubated for a total of 24 hpi. Supernatant and cell lysate samples were collected at 6 hpi, 12 hpi and 24 hpi for determination of virus release and NP expression respectively. To study multiple rounds of infection, the experiment was carried out as above, but the infection was performed using an MOI of 0.01 and supernatant and lysate samples were collected at 24 and 48 hpi. For more detailed analysis, C6/36 (4 × 10^5^ cells/well) were seeded in 12-well plates and incubated at 28 °C. The cells were then infected with rBUNV-WT-Gc-HA or rBUNV-Δ7-Gc-HA at an MOI of 5 and incubated for a total of 24 hpi. Supernatant and lysate samples were collected every 3 hours following infection for determination of virus release and NP and Gc-HA expression respectively.

For collection of intracellular and extracellular virus samples, A549 (1.5 × 10^5^ cells/well) and C6/36 (4 × 10^5^ cells/well) were seeded in 12-well plates, infected with rBUNV-WT or rBUNV-Δ7 at an MOI of 5 and incubated for 24 hours at either 37 °C (mammalian) or 28 °C (insect). Supernatants (extracellular – 1 mL) were collected into cryo-vials and stored at -80 °C. Cells (intracellular) were washed twice with 1 × TNE (10 mM Tris-HCl, pH 7.4, 100 mM NaCl, 1 mM EDTA) and then scraped into 1 mL 1 × TNE and freeze-thawed three times to lyse the cellular membranes. Both extracellular and intracellular samples were titred by crystal violet plaque assay.

To examine BUNV behaviour at lower incubation temperatures, A549 (2 × 10^5^ cells/well) or BHK-21 (2 × 10^5^ cells/well) were seeded in 12-well plates and incubated at 30 °C. The cells were then infected with rBUNV-WT or rBUNV-Δ7 at an MOI of 5 and incubated for a total of 24 hpi. Supernatant samples were collected at 24 hpi for determination of virus titre (*n = 1*).

To determine the effect of mutating the trimerisation residues or the glycosylation site missing in rBUNV-∆7, A549 (1.5 × 10^5^ cells/well) and C6/36 (4 × 10^5^ cells/well) were seeded in 12-well plates, infected with either rBUNV-WT, rBUNV-Δ7, rBUNV-∆tripod or rBUNV-N624Q at an MOI of 1 and incubated for 24 hours at either 37 °C (mammalian) or 28 °C (insect). The MOI was reduced to 1 due to lower titres of the rBUNV-∆tripod and rBUNV-N624Q stocks. Supernatant samples were collected at 24 hpi for determination of virus titre (*n = 2*).

### Mosquito infection

Female *Aedes aegypti* mosquitoes (Poza Rica, Mexico) were sorted and housed in cardboard pint cups the day prior to infection. Following a 24-hour starvation period, the mosquitoes were fed a blood meal of washed sheep’s blood containing either rBUNV-WT or rBUNV-∆7 at 1 × 10^7^ PFU/mL, supplemented with 5 mM ATP. Engorged mosquitoes were transferred to new cups with 10% sucrose and kept at 28 °C and 70% humidity for seven days, at which point legs and wings were harvested in microcentrifuge tubes containing glass beads and 250 μL DMEM supplemented with 2% newborn calf serum (NBCS). Bodies were also collected in 250 μL DMEM with 2% NBCS. Samples were ground using a Pro Series Bullet Blender homogenizer for one minute at maximum speed and then centrifuged at 10,000 *× g* for three minutes. Plaque assays were conducted on all samples to determine viral titres, as follows. Supernatant from each homogenized mosquito sample was serially diluted 10-fold in DMEM and inoculated onto a monolayer of Vero cells. Cells were incubated at 37 °C and 5% CO_2_ for one hour prior to being overlaid with 1.5 mL DMEM supplemented with 0.8% agarose, 2% NBCS, and 1% Antibiotic-Antimycotic. Cells were incubated at 37 °C for three days and then fixed with 4% formalin for one hour before the overlay was removed and cells were stained with crystal violet to allow plaques to be counted. Viral titres were quantified by counting plaques on the lowest countable dilution.

Day 0 samples were collected from entire mosquitoes harvested immediately after feeding on the infectious blood meal. Samples were homogenized and clarified as detailed above. Plaque assays were conducted to determine viral titers.

For protein analysis, entire mosquitoes were harvested in 2 × Laemmli buffer 7 days post-infection and ground using a pestle for western blotting.

### Cholesterol repletion in insect cells

To understand whether the lack of cholesterol in insect cells was affecting rBUNV-∆7 assembly, C6/36 (4 × 10^5^ cells/well) were seeded on glass coverslips in 12-well plates and incubated for 24 hours at 28 °C. Prior to infection, the cells were pre-treated with either methyl-β-cyclodextrin (MBCD; 85 µM; vehicle control), or 0.05 mg/mL or 0.1 mg/mL cholesterol, for 1 hour. The cells were then washed three times with 1 × PBS. Next, rBUNV-WT or rBUNV-∆7 at an MOI of 5 in serum-free Leibovitz’s L-15 media was added to the cells and incubated at 28 °C for 1 hour. The cells were then washed three times with 1 × PBS and incubated for 24 hours at 28 °C in serum-free Leibovitz’s L-15 media. Supernatant samples were collected at 24 hpi for determination of virus titre (*n = 1*).

### Virus purification

T175 flasks were seeded with 2.75 × 10^7^ C6/36 cells at least 24 hours prior to infection. Cells were mock-infected or infected with rBUNV-WT-Gc-HA or rBUNV-∆7-Gc-HA, at a MOI of 0.01, for 1 hour in serum free media at 28 °C, after which complete Leibovitz’s L-15 media was added. At 48 hpi, supernatants were clarified by low-speed centrifugation and filtered (0.45 µm), followed by pelleting through a 20% sucrose cushion by ultracentrifugation. The virus pellet was resuspended in 0.1 × PBS, supplemented with 1 × cOmplete, Mini, EDTA-free Protease I inhibitor cocktail (Sigma-Aldrich) and incubated overnight at 4 °C. The resuspended pellet was aliquoted and stored at -80 °C. Samples were taken for silver stain analysis and western blot analysis.

### Transmission electron microscopy of insect cell slices

C6/36 cells (4 × 10^6^) were seeded in T25 flasks 24 hours prior to infection. Cells were mock-infected or infected with rBUNV-WT or rBUNV-∆7 at an MOI of 5 to ensure every cell was infected. The cells were incubated for 1 h at 28 °C, after which the inoculum was replaced with complete Leibovitz’s L-15 media and incubated for a further 24 hours at 28 °C. Cells were fixed with 2.5% glutaraldehyde in PBS for 2 h at room temperature, pelleted by centrifugation (300 × g, 5 mins), washed twice with PBS and post-fixed with 1% osmium tetroxide in PBS for 1 h. Samples were dehydrated with a graded ethanol series, transitioned through propylene oxide and embedded in Araldite as previously described [44]. Resin blocks were polymerised overnight at 60 °C. Ultra-thin sections were prepared using a Reichert-Jung ultracut E ultramicrotome, mounted on 3.05 mm grids and then stained with saturated uranyl acetate and Reynolds lead citrate as previously described [45].

### Virus titration

Plaque assays were performed on confluent monolayers of BHK-21 cells. Serial dilutions of BUNV (10-fold) made in serum-free DMEM were added to the cells and incubated for 1 h at 37 °C. The dilutions were removed from the cells and overlaid with 1.2% Avicel (RC-581, FMC Biopolymer). After 3 days incubation at 37 °C, the cells were fixed through the overlay with 8% formaldehyde in water for 10 mins. The fixative was washed off with water and then the cells were stained with 2% crystal violet in 20% ethanol. Plaques were counted and the titre was determined.

### Western blot analysis

Cells were lysed with 1 × radioimmunoprecipitation assay (RIPA) buffer (50 mM Tris-HCl pH 7.5, 150 mM NaCl, 1% (v/v) NP40 alternative, 0.5% (w/v) sodium deoxycholate and 0.1% sodium dodecyl sulphate (SDS; w/v)), supplemented with 1  × Halt Protease Inhibitor Cocktail (100 × ; Thermo Fisher Scientific) for 15 min on ice. Lysates were collected; proteins were resolved on a 12% or 15% (immunoprecipitation samples) SDS-PAGE gel and then transferred to a polyvinylidene fluoride (PVDF) membrane. The transfer was performed at 15 V for 30 min, using the Trans-Blot turbo (Bio-Rad). After transfer, the membrane was blocked for 1 h in Odyssey blocking buffer (PBS) (Licor; diluted 1:1 with 1 × PBS containing 0.1% Tween 20 [PBS-T]). Subsequently, the membrane was stained with the primary antibodies (made in 1:4 [blocking buffer: PBS-T]; anti-BUNV NP 1:5000, anti-actin 1:5000, anti-HA 1:5000) for 1 h rocking, at room temperature and then with corresponding secondary antibodies (made in 1:4 [blocking buffer: PBS-T]; all secondary antibodies used at 1:10000) for 1 h at room temperature. The membrane was washed and visualised on the Odyssey M imaging system (Licor). Densitometry analysis was performed using ImageJ over three independent experiments.

### Silver stain analysis

Viral pellet samples (10 µL) were resolved by 15% SDS-PAGE and then fixed and stained using the ProteoSilver Kit (Sigma-Aldrich), following the manufacturer’s protocol.

### RNA extraction and reverse transcription - quantitative PCR (RT-qPCR)

Culture supernatants (1 mL) were collected at 24 h post-infection (hpi) from C6/36 cells that were mock-infected or infected with rBUNV-WT-Gc-HA or rBUNV-Δ7-Gc-HA (n = 3). Total RNA was extracted from supernatants using the Monarch Total RNA Miniprep Kit according to the manufacturer’s instructions, including the on-column DNase I treatment. The eluted RNA was subjected to an additional in-solution DNase I treatment and subsequently re-purified using the RNA purification columns provided with the kit. RNA was eluted in 23 µL of nuclease-free water.

For complementary DNA (cDNA) synthesis, 10 µL of purified RNA was reverse-transcribed using the LunaScript RT SuperMix Kit, which contains random hexamer primers, according to the manufacturer’s instructions. Quantitative PCR was performed using the Luna Universal qPCR Master Mix on the Stratagene Mx3005P qPCR system (Agilent Technologies) with primers specific for the BUNV S segment (forward: [CACACCACTGGGCTTAGTTAT, 5′–3′]; reverse: [AGTGTAACTTCCCATTCACTTCT, 5′–3′]). Viral RNA copy numbers were determined by absolute quantification using the cycle threshold (Ct) method.

Due to a lack of endogenous reference transcript present in cell-free supernatants, a standard curve was generated from *in vitro*-transcribed BUNV S segment RNA. Briefly, the pT7riboBUNS(+) plasmid was used as a template for transcription with the HiScribe T7 Quick High Yield RNA Synthesis Kit (NEB) according to the manufacturer’s instructions. Serial dilutions of the resulting RNA transcript were used to generate a standard curve for calculation of viral RNA copy numbers in experimental samples. Only amplification signals with Ct values ≤25 were included in the analysis.

### Immunofluorescence analysis

To assess localisation of BUNV proteins during the virus replication cycle, C6/36 cells (4  × 10^5^) were grown on glass coverslips and infected with rBUNV-WT-Gc-HA (MOI = 5) or rBUNV-Δ7-Gc-HA (MOI = 5). At specified time points, the cells were fixed with 4% formaldehyde for 10 min, stained with concanavelin A (5 µg/mL) for 10 min, permeabilised with 0.05% triton-x-100 for 10 min and blocked with 1% bovine serum albumin (BSA) for 1 h. Cells were then labelled with anti-BUNV NP (1:5000) and anti-HA (for Gc staining; 1:5000) for 1 h, followed by labelling with corresponding Alexa Fluor 488 nm or 647 nm secondary antibodies (all secondary antibodies used at 1:5000), respectively, for 1 h. Cells were then washed and mounted onto microscope slides using EverBrite mounting media containing DAPI (Biotium). Stained cells were imaged on a Zeiss LSM 990 confocal microscope at 63 × magnification. The images were processed on Fiji [46]. In order to generate the membrane mask, used in subsequent statistical analysis, a binary image was created whereby the threshold was adjusted to flood the cells with membrane signal and then the MorphoLibJ plugin was used to detect the edges of the cells and create the plasma membrane mask with a 4 pixel internal gradient added [47].

For non-permeabilised samples, all steps were performed as above, with the membrane staining and permeabilization steps omitted, and the images were collected on the Olympus IX83 widefield microscope at 100 × magnification.

### Immunoprecipitation of Gc-HA and interacting proteins

A549 or C6/36 cells (1 × 10^7^ cells/flask) were seeded and incubated at either 37 °C (mammalian) or 28 °C (insect). The cells were then infected with rBUNV-WT-Gc-HA or rBUNV-Δ7-Gc-HA at an MOI of 1 and incubated for a total of 24 hpi. The MOI was changed to 1 to reduce the required amount of the rBUNV-WT-HA and rBUNV-∆7-HA stocks due to the lower titres obtained. Cells were washed once with 1 × PBS and lysed in 1 mL immunoprecipitation lysis buffer (25 mM Tris-HCl, 150 mM NaCl, 1 mM EDTA, 1% NP-40, 5% glycerol), supplemented with 1 × Halt Protease Inhibitor Cocktail (100 × ; Thermo Fisher Scientific). The cells were lysed for 30 min at 4 °C, after which the lysates were collected and centrifuged at 13,000 *× g* for 15 min. The lysates were incubated rotating overnight at 4 °C with Dynabead Protein G beads, which had previously been incubated with anti-HA antibody (1:80) in PBS-T (0.02% Tween 20) for 2 h at room temperature and then washed with PBS-T to remove any unbound antibody. After overnight incubation, the beads were drawn out of solution using the DynaMag magnetic rack (Invitrogen), and unbound protein was removed. The beads were subsequently washed three times with PBS-T and then resuspended in 50 µL PBS-T for mass spectrometry analysis.

### TMT labelling and high-pH reversed phase (RP) chromatography

Mammalian and insect samples were analysed in separate TMTpro 16plex experiments. Immuno-isolated samples were reduced with TCEP, alkylated with iodoacetamide and digested from the beads with trypsin overnight at 37 °C. Peptides were labelled with TMTpro 16plex reagents according to the manufacturer’s protocol (Thermo Fisher Scientific), pooled and desalted using SepPak cartridges following the manufacturer’s instructions (Waters). Labelled peptides were fractionated by high-pH reversed-phase chromatography using an Ultimate 3000 liquid chromatography system (Thermo Scientific). Samples were loaded onto an XBridge BEH C18 Column (Waters) and peptides were eluted with a 0–95% acetonitrile gradient in 20 mM ammonium hydroxide (pH 10) over 60 minutes. Resulting fractions were concatenated into four pools, dried and resuspended in 1% formic acid prior to analysis by nano-LC MS/MS.

### Nano-LC mass spectrometry

Peptides were separated using an Ultimate 3000 nano-LC system in line with an Orbitrap Fusion Tribrid mass spectrometer (Thermo Scientific). Samples were loaded onto an Acclaim PepMap C18 nano-trap column (Thermo Scientific) and resolved on an Acclaim PepMap C18 reverse phase analytical column (Thermo Scientific) over a 150 min gradient at 300 nl min − 1. All spectra were acquired in data-dependent SPS-MS3 mode using standard settings for TMT quantification. FTMS1 spectra were collected at 120,000 resolution and FTMS3 spectra were collected at 50,000 resolution.

### Mass spectrometry data analysis

Raw data files were processed in Proteome Discoverer (Thermo Scientific) using the SEQUEST HT algorithm, and searched against the UniProt human or *Aedes albopictus* proteomes, together with the UniProt *Bunyamwera virus* database. Searches allowed a maximum of two missed tryptic cleavages, with carbamidomethylation of cysteine and TMT modification of peptide N-termini and lysine residues as fixed modifications, and methionine oxidation and protein N-terminal modifications as variable modifications. Peptide and protein identifications were filtered to a 5% false discovery rate (FDR).

### Mass spectrometry statistical analysis

Protein abundances were normalised by total peptide abundance before being log_2_ transformed. Additional normalisation to the abundance of common Gc peptides shared between the WT and ∆7 samples was included to account for differences in Gc concentration between the two samples. Statistical analyses were performed in R using the limma package [48,49]. A single linear model was fitted across all conditions and moderated t-tests were performed for each comparison of interest with Benjamini-Hochberg FDR correction for multiple testing. PCAs were calculated using the prcomp function. This confirmed that there was consistency across biological repeats and the samples clustered together within different repeats.

### Mass spectrometry volcano plots

For each comparison, log_2_ fold change was plotted against the negative log_10_ adjusted p-value. Proteins where p < 0.05 were highlighted in yellow and proteins where p < 0.01 were highlighted in green. Proteins where p < 0.05 and Log_2_FC>1, or where p < 0.05 and Log_2_FC < -1 were highlighted in red.

### Statistical analysis

The statistical significance of data was determined by performing a Student’s *t* test. The statistical significance of the titre data from mosquitoes was determined by performing a Mann Whitney test. The statistical significance of the quantified RT-qPCR data from supernatants was Log_10_-transformed and analysed by Welch’s unpaired t-test, setting the samples which had no detectable data the value of the limit of detection. Significance was deemed when the values were less than or equal to the 0.05 *p* value.

## Supporting information

S1 Fig Cartoon depicting the location of naturally occurring deletions relative to the BUNV-∆7 deletion. Depiction of the M segments of Maguari virus (MAGV; A), Schmallenberg virus (SBV; B) and Bunyamwera orthobunyavirus (BUNV; C), indicating the amino acid numbers of the start and end positions of the Gn, NSm and Gc genes. Previously described deletion mutants have been included, with the amino acid numbers representing start and end positions of the deletions. Non-temperature sensitive (non-ts) R1 and R2 MAGV were identified by passaging MAGV through A549 cells. The hypervariable region (HVR) of SBV has been depicted, alongside natural isolates BH04/15 and BH19/14. BUNV-∆7 is a deletion mutant identified by creating serial deletions using a reverse genetics system.
(TIF)

S2 Fig Recovery of wildtype BUNV and ∆7 BUNV from plasmid. (A) Schematic depiction of plasmid pT7riboBUNM(+) encoding the M segment of BUNV, with the indication of the ∆7 deletion (479 AA – 827 AA inclusive) from Gc. The Gn, NSm and Gc genes have been shown, flanked by the BUNV M untranslated regions (UTR), hepatitis delta virus ribozyme (Rz) and T7 promoter (T7P) and terminator (T7T) sequences. (B) Lysates were collected from BSR-T7 cells transfected with the BUNV rescue plasmids 5 days post transfection. Supernatant at this point was collected and transferred to BHK cells and lysates were collected 2 days post infection. Appropriate controls were included whereby pT7riboBUNL(+) had been excluded to prevent rescue of infectious virus (-L). The lysates were probed for NP expression and actin expression, as a loading control, using specific antisera as indicated.
(TIF)

S3 Fig Comparison of multiple replication cycles of wildtype BUNV and ∆7 BUNV in A549 and C6/36 cells. A549 cells (mammalian) and C6/36 cells (insect) were either mock-infected or infected with rBUNV-WT (WT) or mutant rBUNV-∆7 (∆7) at an MOI of 0.01 and supernatants were collected at either 24 or 48 hours post infection (n = 3). (A) Titration of the supernatants was performed by plaque assay. Log_10_-transformed samples were analysed by Student’s t-test (unpaired) whereby n.s. = p > 0.05, *** = p < 0.0001, comparing WT to ∆7 at each timepoint.
(TIF)

S4 Fig Recovery of wildtype BUNV and ∆7 BUNV with HA-tagged Gc from plasmid. (A) Schematic depiction of plasmids pT7riboBUNM-HA(+) and pT7riboBUNMΔ7-HA(+) encoding the BUNV WT or ∆7 M segment and indicating the approximate site of the HA tag insertion in both plasmids. The Gn, NSm and Gc genes have been shown, flanked by the BUNV M untranslated regions (UTR), hepatitis delta virus ribozyme (Rz) and T7 promoter (T7P) and terminator (T7T) sequences. (B) Lysates collected from BSR-T7 cells at 5 days post transfection and BHK cells at 2 days post infection were probed for HA, NP and actin, as a loading control, expression. Appropriate controls were included whereby pT7riboBUNL(+) had been excluded to prevent rescue of infectious virus (-L).
(TIF)

S5 Fig Transmission electron microscopy images of C6/36 cells infected with wildtype BUNV or ∆7 BUNV. Electron microscopy of mock-infected C6/36 cells (A) or C6/36 cells infected with wildtype BUNV (B) or mutant ∆7 BUNV (C). Zoomed images are shown on the right, indicated in the non-zoomed image by a white dashed box. Scale bars represent 2 µm in the right images and 200 nm in the left images.
(TIF)

S6 Fig Investigation of the differences between mammalian and insect cell lines and the effect on wildtype BUNV and ∆7 BUNV. (A) A549 and BHK cells were incubated at 30 °C to reflect the incubation temperature of C6/36 cells. The cells were then infected with wildtype BUNV (WT; red) and ∆7 BUNV (∆7; blue) and incubated at 30 °C for 24 hours. Supernatant was collected and titred by plaque assay (n = 1). (B) C6/36 cells were pre-treated with either H_2_O (grey), methyl-β-cyclodextrin (MBCD; 85 µM; vehicle control; dark green), 0.1 mg/mL cholesterol (green) or 0.05 mg/mL cholesterol (light green), for 1 hour. The cells were then infected with wildtype BUNV (WT) or mutant ∆7 BUNV (∆7) at an MOI of 5. Supernatant was harvested at 24 hours post infection and titred by plaque assay (n = 1). (C) A549 and C6/36 cells were infected at an MOI of 5 with either wildtype BUNV (WT; red), mutant ∆7 BUNV (∆7; blue), BUNV containing a mutated glycosylation site (N624; pink) or BUNV lacking trimerisation residues in the Gc head (dark blue). The supernatant was collected at 24 hours post infection and was titred by plaque assay (n = 2). All titres were plotted on a logarithmic scale graph.
(TIF)

S7 Fig Confocal microscopy images of C6/36 cells infected with wildtype rBUNV-HA or ∆7 rBUNV-HA at 24 hours post infection. C6/36 cells were infected with rBUNV-WT-Gc-HA (A) or rBUNV-∆7-Gc-HA (B) at an MOI of 5 and fixed with formaldehyde at 24 hpi. The cells were then permeabilized, blocked, and stained for the nucleus (DAPI, blue), concanavalin A (membrane, cyan), BUNV NP (green), and BUNV Gc-HA (red) by indirect immunostaining. The cells were imaged on LSM990 confocal microscope at ×63 magnification. Scale bars representing 20 µm are shown. DAPI, 4,’6-diamidino-2-phenylindole. (C) Co-occurrence analysis was performed by analysing the proportion of Gc signal or NP signal that was present in the same area as a mask representing the plasma membrane of the cells. Approximately 20 cells were selected for each condition to be analysed. All results were analysed by Student’s t-test (unpaired) whereby n.s. = p > 0.05; ** = p < 0.01, comparing WT-HA to ∆7-HA for each protein.
(TIF)

S8 Fig Immunoprecipitation of wildtype rBUNV-Gc-HA and ∆7 rBUNV-Gc-HA from A549 and C6/36 cells. A549 or C6/36 cells were infected with either rBUNV-WT-Gc-HA (WT-HA; red) or rBUNV-∆7-Gc-HA (∆7-HA; blue) and incubated for 24 hours. Cell lysates were collected and incubated with magnetic IgG beads, pre-bound to anti-HA antibody. The beads were washed several times and then both the original lysates (A) and the beads (B) were analysed by western blot analysis, probing for expression of HA and NP. (C) Densitometry analysis performed on (B) as expression of NP relative to Gc-HA. The expression of BUNV NP was normalised to rBUNV-WT-Gc-HA from each cell line (n = 3). All results were analysed by Student’s t-test (unpaired) whereby n.s. = p > 0.05; ** = p < 0.01, comparing WT-HA to ∆7-HA for each cell line.
(TIF)

S9 Fig Uncropped western blots from Fig 1A; Comparison of single cycle growth kinetics between wildtype BUNV and ∆7 BUNV in multiple cell lines. Uncropped western blots from A549 cells (panels 1 and 2) and BHK cells (panels 3 and 4), whereby lysates were analysed for NP and actin expression, at 6-, 12- and 24 hours post infection with rBUNV-WT (WT) or mutant rBUNV-∆7 (∆7) at an MOI of 5.
(PDF)

S10 Fig Uncropped western blots from Fig 1A; Comparison of single cycle growth kinetics between wildtype BUNV and ∆7 BUNV in multiple cell lines. Uncropped western blots from C6/36 cells (panels 5 and 6), whereby lysates were analysed separately for NP and actin expression, at 6-, 12- and 24 hours post infection with rBUNV-WT (WT) or mutant rBUNV-∆7 (∆7) at an MOI of 5.
(PDF)

S11 Fig Uncropped western blots from Fig 2B; Comparison of infection of *Aedes* mosquitoes by wildtype BUNV and ∆7 BUNV. Uncropped western blots from *Aedes* mosquitoes, which were fed a blood meal containing 1x10^7^ pfu/mL of rBUNV-WT or rBUNV-∆7. Mosquito lysates (10 mosquitoes for each virus; panels 1–2 represent first five mosquitoes for each virus and panels 3–4 represent second five mosquitoes for each virus) were subject to western blot analysis and probed for expression of NP and actin, as a loading control.
(PDF)

S12 Fig Uncropped western blots from Fig 3A; Comparison of growth kinetics and protein expression of wildtype rBUNV-HA and ∆7 rBUNV-HA in C6/36 cells. Uncropped western blots from C6/36 cells, whereby lysates were analysed for NP and actin expression and the western blot was cut to analyze for HA expression separately, at every 3 hours post infection until 24 hpi with rBUNV-WT-Gc-HA (WT-HA; panels 1–3) or mutant rBUNV-∆7-Gc-HA (∆7-HA; panels 4–6) at an MOI of 5.
(PDF)

S13 Fig Uncropped gel and western blot from Fig 4C-D; Comparison of intracellular and extracellular virus production of wildtype BUNV and ∆7 BUNV in A549 and C6/36 cells. Uncropped silver stain (C) and western blot (D) from C6/36 cellular purified supernatant, which had been previously infected with rBUNV-WT-Gc-HA or rBUNV-∆7-Gc-HA. The resuspended pellet was subject to silver stain analysis (C) and western blot analysis (D), probing for expression of HA and NP. The protein ladder sizes are indicated (kDa) as well as the predicted bands for viral structural proteins WT-Gc, ∆7-Gc, Gn and NP.
(PDF)

S14 Fig Uncropped western blot from S2B Fig; Recovery of wildtype BUNV and ∆7 BUNV from plasmid. Uncropped western blots from lysates collected from BSR-T7 cells transfected with the BUNV rescue plasmids 5 days post transfection and BHK infected with supernatant from the BSR-T7 cells and incubated 2 days (B). Appropriate controls were included whereby pT7riboBUNL(+) had been excluded to prevent rescue of infectious virus (-L). The lysates were probed for NP expression and actin expression, as a loading control.
(PDF)

S15 Fig Uncropped western blot from S4B Fig; Recovery of wildtype BUNV and ∆7 BUNV with HA-tagged Gc from plasmid. Uncropped western blots from lysates collected from BSR-T7 cells at 5 days post transfection with pT7riboBUNM-HA(+) and pT7riboBUNMΔ7-HA(+) (alongside other BUNV segment-expressing plasmids) and BHK cells at 2 days post infection. The lysates were probed for NP and actin expression, and the western blot was cut to analyze for HA expression separately. Appropriate controls were included whereby pT7riboBUNL(+) had been excluded to prevent rescue of infectious virus (-L).
(PDF)

S16 Fig Uncropped western blots from S8A Fig; Immunoprecipitation of wildtype rBUNV-Gc-HA and ∆7 rBUNV-Gc-HA from A549 and C6/36 cells. Uncropped western blots from lysates collected from A549 or C6/36 cells 24 hours post infection with either rBUNV-WT-Gc-HA (WT-HA) or rBUNV-∆7-Gc-HA (∆7-HA). The lysates (panels 1–4) were probed for expression of HA and NP, independently on western blots that were cut.
(PDF)

S17 Fig Uncropped western blots from S8B Fig; Immunoprecipitation of wildtype rBUNV-Gc-HA and ∆7 rBUNV-Gc-HA from A549 and C6/36 cells. Uncropped western blots from immunoprecipitations beads, which had been incubated with lysates collected from A549 or C6/36 cells 24 hours post infection with either rBUNV-WT-Gc-HA (WT-HA) or rBUNV-∆7-Gc-HA (∆7-HA). The beads (panels 1–4) were washed and probed for expression of HA and NP, independently on western blots that were cut.
(PDF)

S1 Data Mass spectrometry analysis dataset of proteins identified from immunoprecipitation with BUNV Gc-HA in A549 cells or C6/36 cells. (XLSX)
