# Supplementary material for: The Bunyamwera orthobunyavirus Gc glycoprotein head and stalk drives an infectious virion assembly pathway specific for the insect host
Source: PLoS Pathog. 2026 Jul 7;22(7):e1014374. doi: 10.1371/journal.ppat.1014374 (PMC13399505; doi:10.1371/journal.ppat.1014374)

SUPP FIG 9 Uncropped western blots from Figure 1A; Comparison of single cycle growth kinetics between wildtype BUNV and Δ7 BUNV in multiple cell lines.

A – Panels 1-2

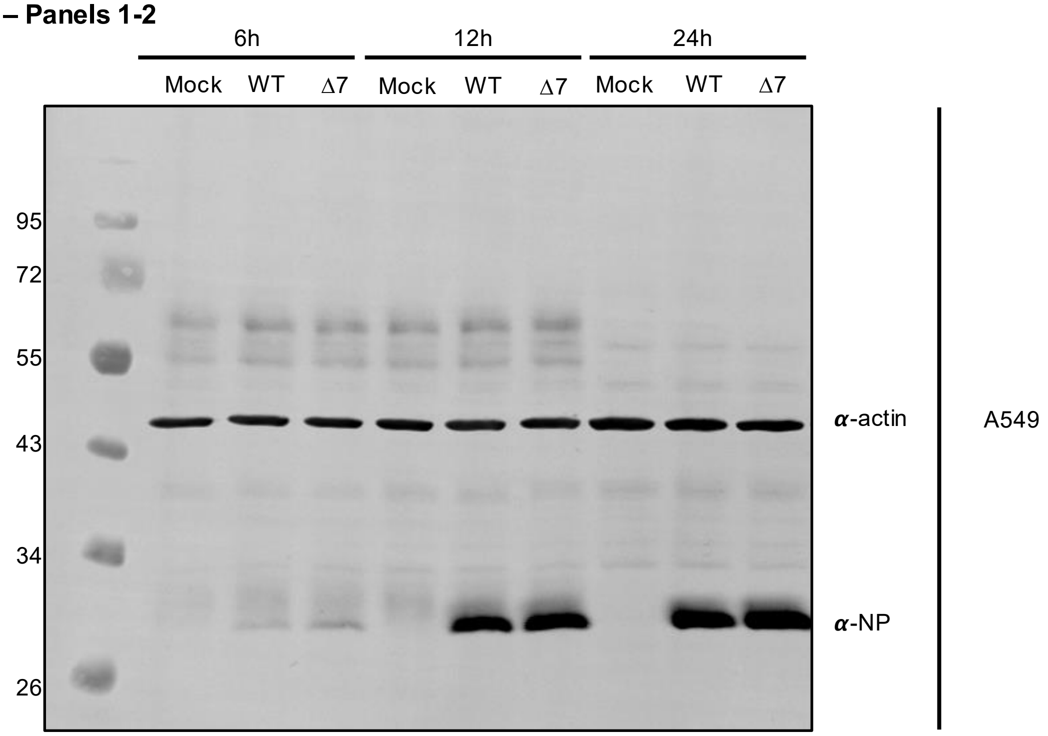

A – Panels 3-4

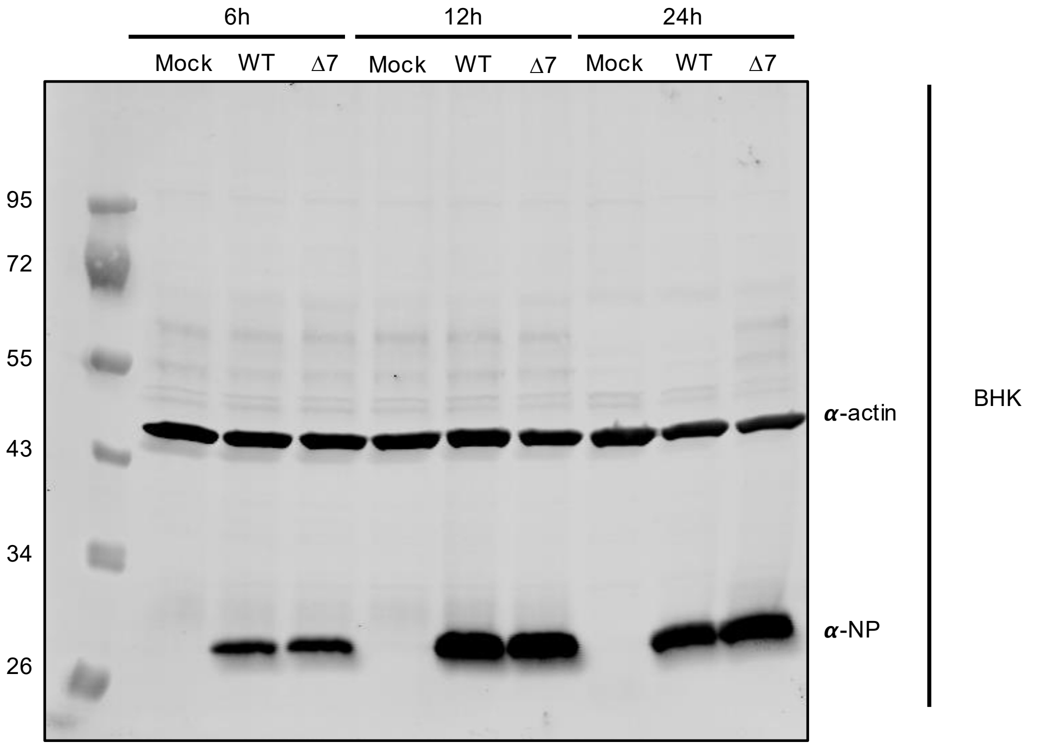

Supplement: S9 Fig — Uncropped western blots from A549 cells (panels 1 and 2) and BHK cells (panels 3 and 4), whereby lysates were analysed for NP and actin expression, at 6-, 12- and 24 hours post infection with rBUNV-WT (WT) or mutant rBUNV-∆7 (∆7) at an MOI of 5. (PDF) [file ppat.1014374.s009.pdf]
