# Supplementary material for: The Bunyamwera orthobunyavirus Gc glycoprotein head and stalk drives an infectious virion assembly pathway specific for the insect host
Source: PLoS Pathog. 2026 Jul 7;22(7):e1014374. doi: 10.1371/journal.ppat.1014374 (PMC13399505; doi:10.1371/journal.ppat.1014374)

SUPP FIG 10 Uncropped western blots from Figure 1A; Comparison of single cycle growth kinetics between wildtype BUNV and Δ7 BUNV in multiple cell lines.

A – Panels 5-6 – actin staining

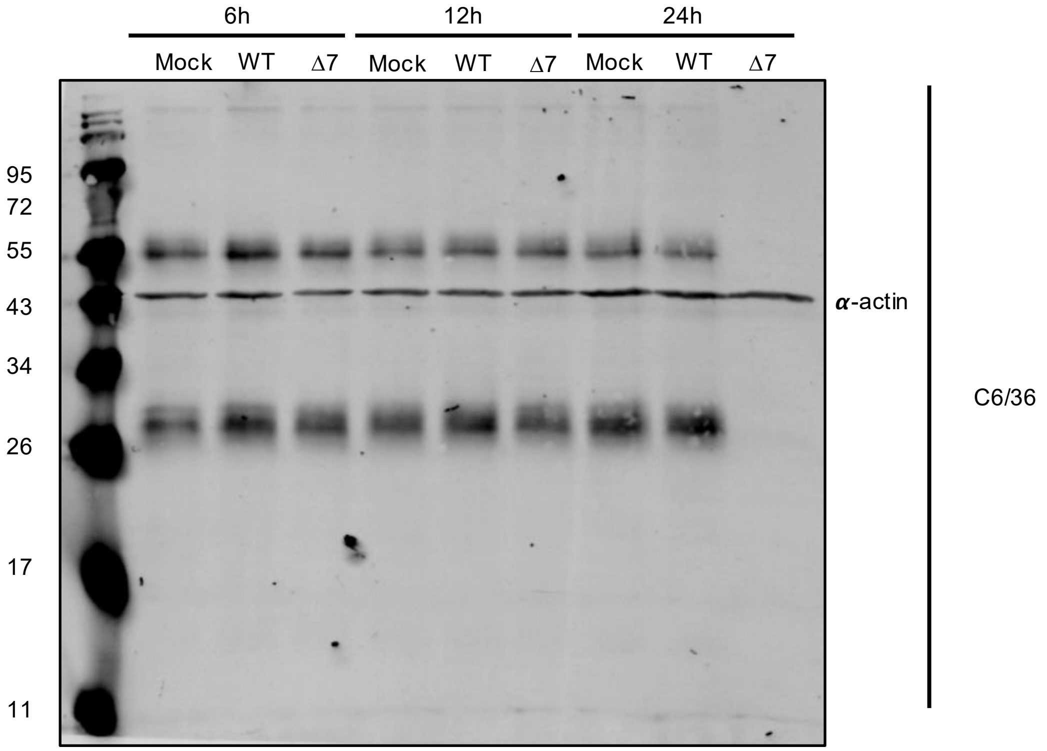

A – Panels 5-6 – NP staining

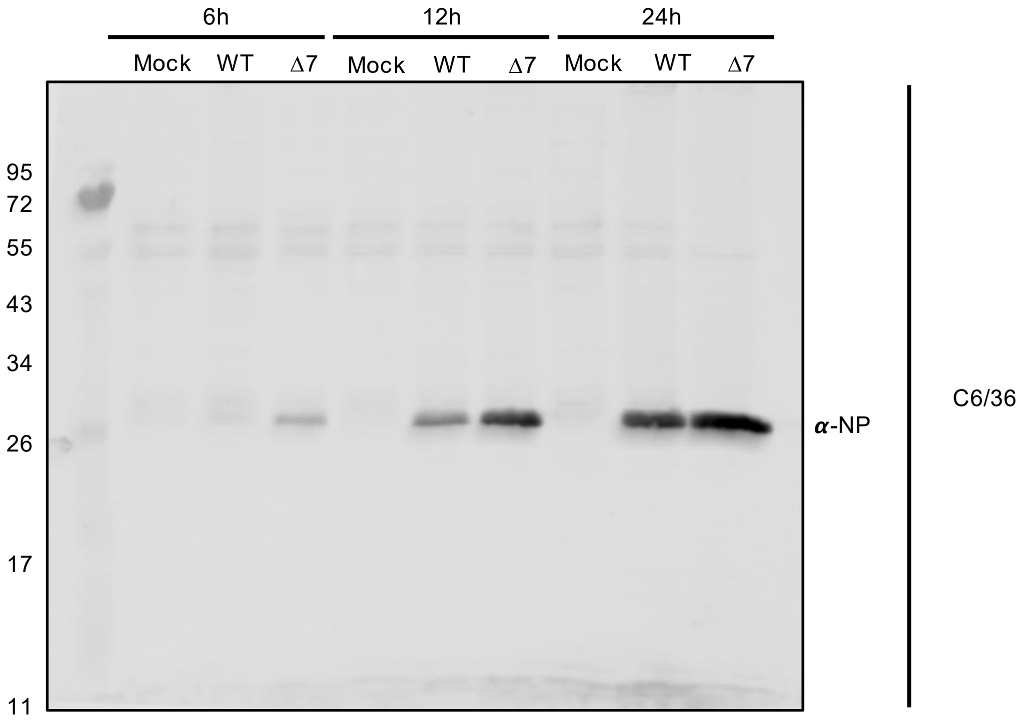

Supplement: S10 Fig — Uncropped western blots from C6/36 cells (panels 5 and 6), whereby lysates were analysed separately for NP and actin expression, at 6-, 12- and 24 hours post infection with rBUNV-WT (WT) or mutant rBUNV-∆7 (∆7) at an MOI of 5. (PDF) [file ppat.1014374.s010.pdf]
