# Supplementary material for: The Bunyamwera orthobunyavirus Gc glycoprotein head and stalk drives an infectious virion assembly pathway specific for the insect host
Source: PLoS Pathog. 2026 Jul 7;22(7):e1014374. doi: 10.1371/journal.ppat.1014374 (PMC13399505; doi:10.1371/journal.ppat.1014374)

SUPP FIG 11. Uncropped western blots from Figure 2B; Comparison of infection of *Aedes* mosquitoes by wildtype BUNV and Δ7 BUNV.

B – Panels 1-2

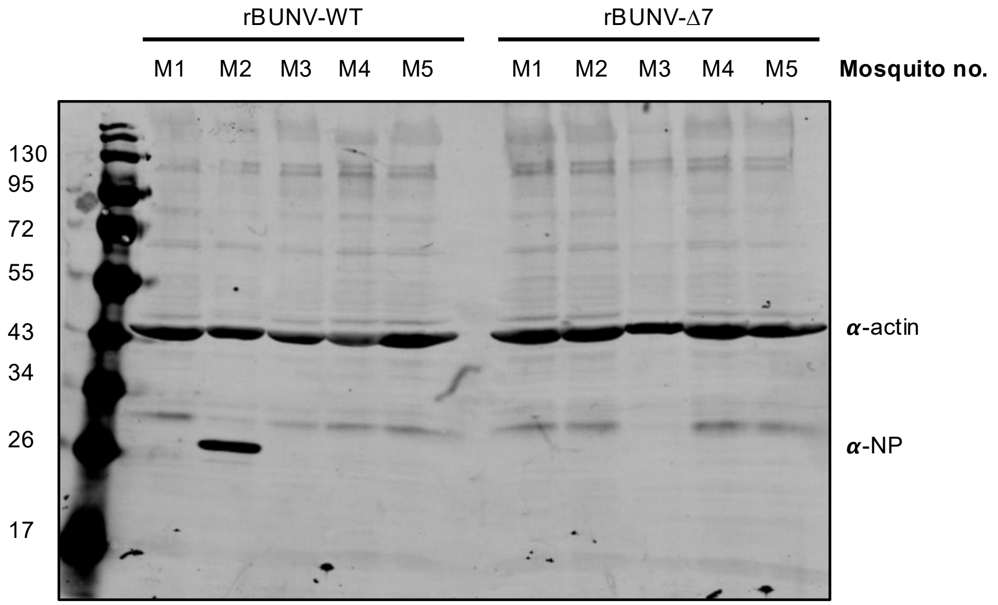

B – Panels 3-4

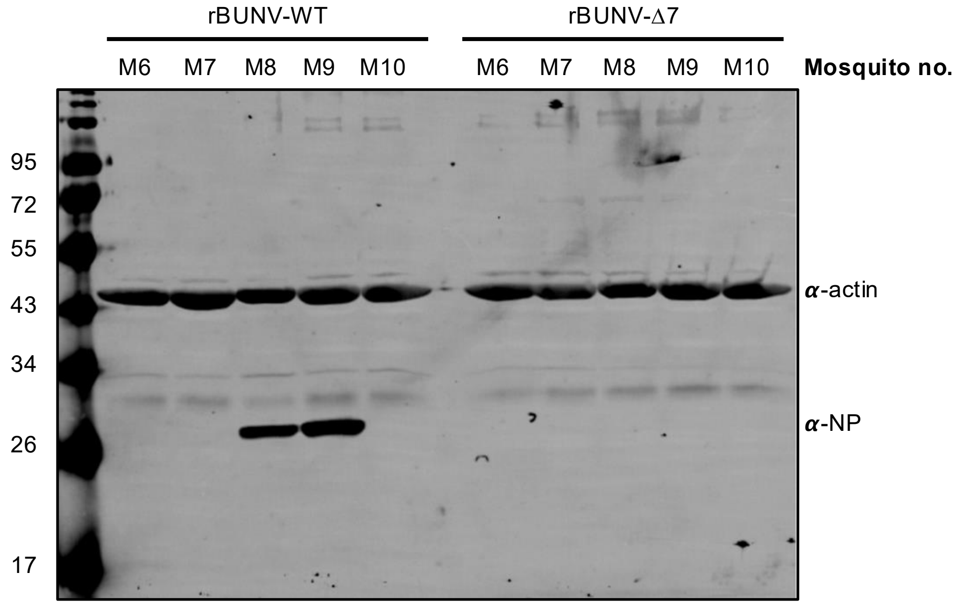

Supplement: S11 Fig — Uncropped western blots from Aedes mosquitoes, which were fed a blood meal containing 1x10^7^ pfu/mL of rBUNV-WT or rBUNV-∆7. Mosquito lysates (10 mosquitoes for each virus; panels 1–2 represent first five mosquitoes for each virus and panels 3–4 represent second five mosquitoes for each virus) were subject to western blot analysis and probed for expression of NP and actin, as a loading control. (PDF) [file ppat.1014374.s011.pdf]
