# Supplementary material for: The Bunyamwera orthobunyavirus Gc glycoprotein head and stalk drives an infectious virion assembly pathway specific for the insect host
Source: PLoS Pathog. 2026 Jul 7;22(7):e1014374. doi: 10.1371/journal.ppat.1014374 (PMC13399505; doi:10.1371/journal.ppat.1014374)

SUPP FIG 12 Uncropped western blots from Figure 3A; Comparison of growth kinetics and protein expression of wildtype rBUNV-HA and ∆7 rBUNV-HA in C6/36 cells.

A – Panels 1-3 – Western cut and probed separately for HA and NP/actin

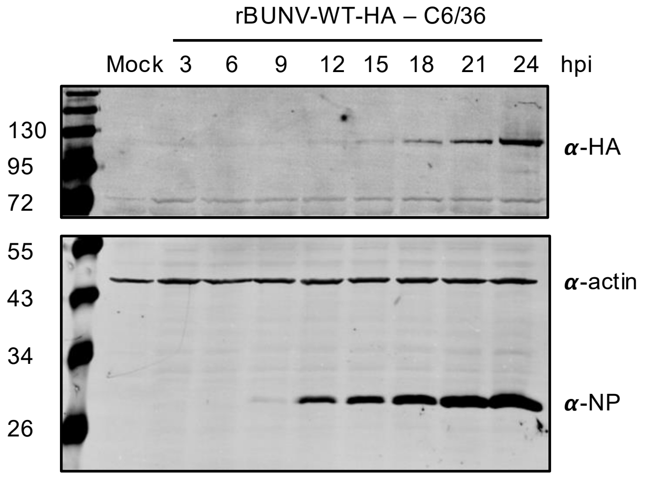

A – Panels 4-6 – Western cut and probed separately for HA and NP/actin

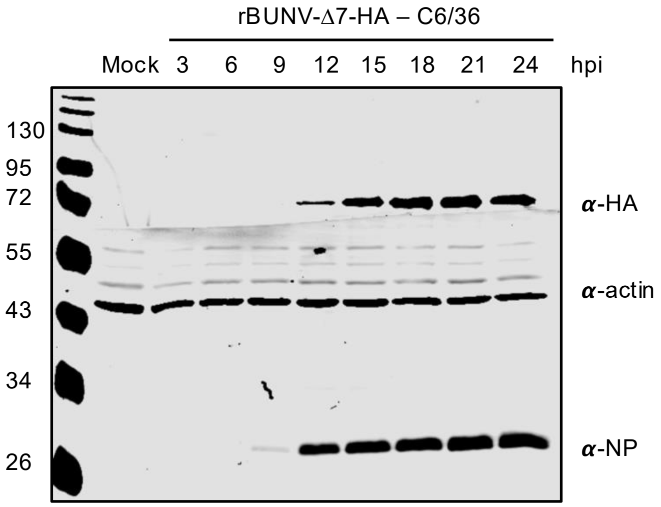

Supplement: S12 Fig — Uncropped western blots from C6/36 cells, whereby lysates were analysed for NP and actin expression and the western blot was cut to analyze for HA expression separately, at every 3 hours post infection until 24 hpi with rBUNV-WT-Gc-HA (WT-HA; panels 1–3) or mutant rBUNV-∆7-Gc-HA (∆7-HA; panels 4–6) at an MOI of 5. (PDF) [file ppat.1014374.s012.pdf]
