# Supplementary material for: The Bunyamwera orthobunyavirus Gc glycoprotein head and stalk drives an infectious virion assembly pathway specific for the insect host
Source: PLoS Pathog. 2026 Jul 7;22(7):e1014374. doi: 10.1371/journal.ppat.1014374 (PMC13399505; doi:10.1371/journal.ppat.1014374)

SUPP FIG 13. Uncropped gel and western blot from Figure 4C-D; Comparison of intracellular and extracellular virus production of wildtype BUNV and Δ7 BUNV in A549 and C6/36 cells.

C

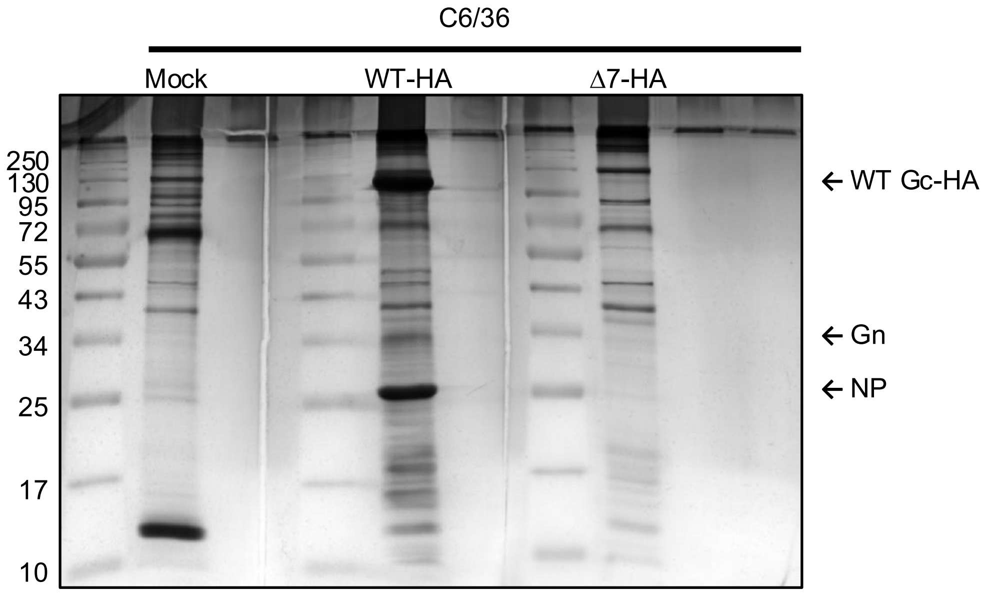

D

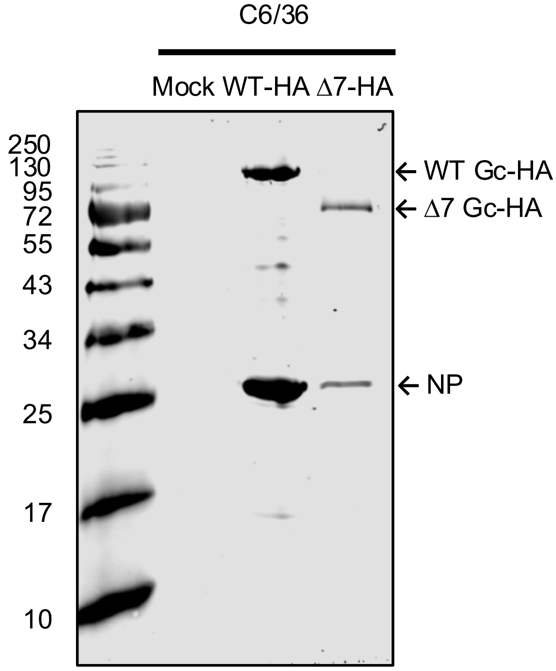

Supplement: S13 Fig — Uncropped silver stain (C) and western blot (D) from C6/36 cellular purified supernatant, which had been previously infected with rBUNV-WT-Gc-HA or rBUNV-∆7-Gc-HA. The resuspended pellet was subject to silver stain analysis (C) and western blot analysis (D), probing for expression of HA and NP. The protein ladder sizes are indicated (kDa) as well as the predicted bands for viral structural proteins WT-Gc, ∆7-Gc, Gn and NP. (PDF) [file ppat.1014374.s013.pdf]
