# Supplementary material for: The Bunyamwera orthobunyavirus Gc glycoprotein head and stalk drives an infectious virion assembly pathway specific for the insect host
Source: PLoS Pathog. 2026 Jul 7;22(7):e1014374. doi: 10.1371/journal.ppat.1014374 (PMC13399505; doi:10.1371/journal.ppat.1014374)

SUPP FIG 14 Uncropped western blot from Supplemental Figure 2B; Recovery of wildtype BUNV and Δ7 BUNV from plasmid.

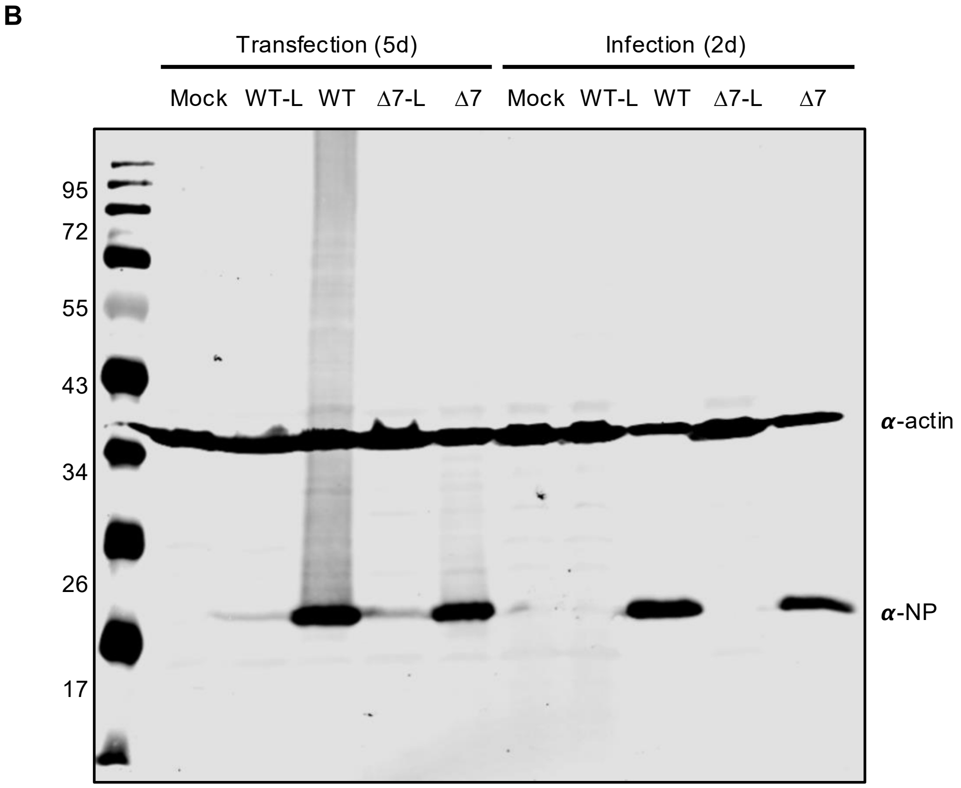

Supplement: S14 Fig — Uncropped western blots from lysates collected from BSR-T7 cells transfected with the BUNV rescue plasmids 5 days post transfection and BHK infected with supernatant from the BSR-T7 cells and incubated 2 days (B). Appropriate controls were included whereby pT7riboBUNL(+) had been excluded to prevent rescue of infectious virus (-L). The lysates were probed for NP expression and actin expression, as a loading control. (PDF) [file ppat.1014374.s014.pdf]
