# Supplementary material for: The Bunyamwera orthobunyavirus Gc glycoprotein head and stalk drives an infectious virion assembly pathway specific for the insect host
Source: PLoS Pathog. 2026 Jul 7;22(7):e1014374. doi: 10.1371/journal.ppat.1014374 (PMC13399505; doi:10.1371/journal.ppat.1014374)

SUPP FIG 15 Uncropped western blot from Supplemental Figure 4B; Recovery of wildtype BUNV and Δ7 BUNV with HA-tagged Gc from plasmid.

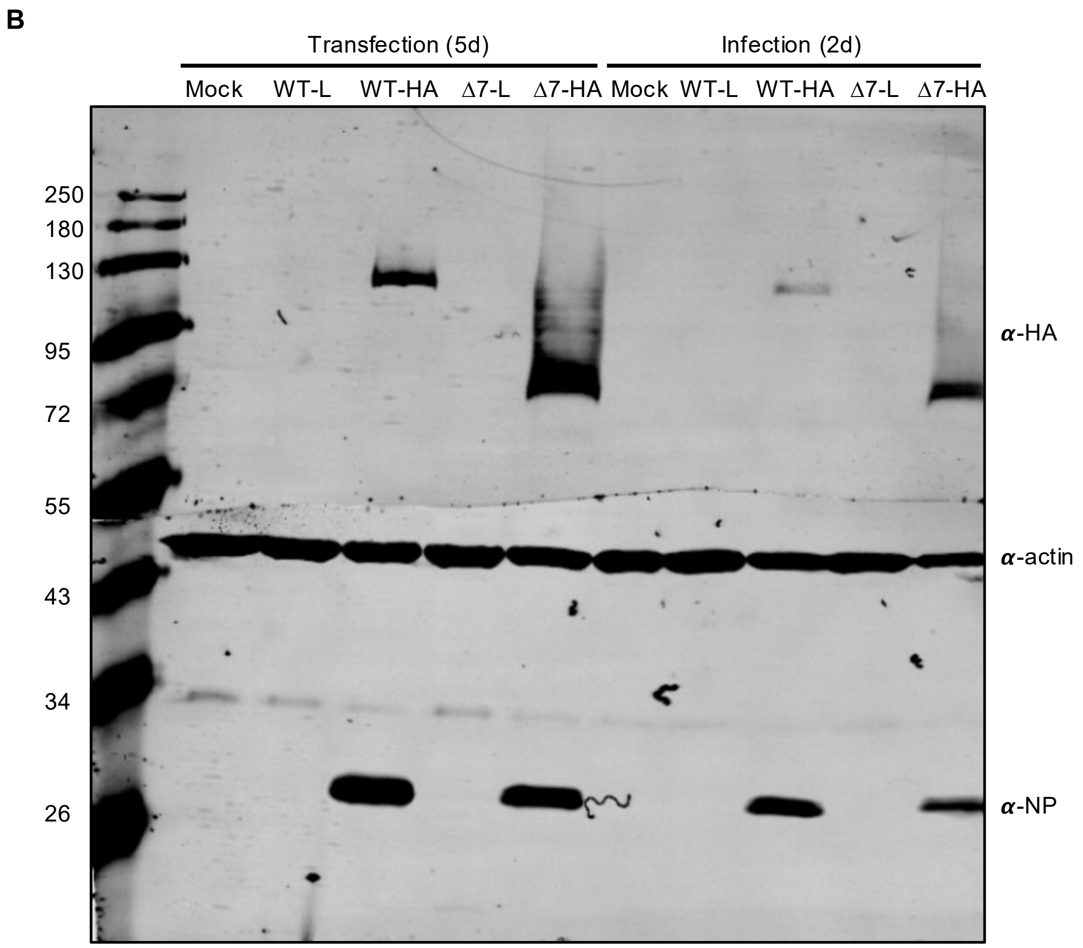

Supplement: S15 Fig — Uncropped western blots from lysates collected from BSR-T7 cells at 5 days post transfection with pT7riboBUNM-HA(+) and pT7riboBUNMΔ7-HA(+) (alongside other BUNV segment-expressing plasmids) and BHK cells at 2 days post infection. The lysates were probed for NP and actin expression, and the western blot was cut to analyze for HA expression separately. Appropriate controls were included whereby pT7riboBUNL(+) had been excluded to prevent rescue of infectious virus (-L). (PDF) [file ppat.1014374.s015.pdf]
