# Supplementary material for: The Bunyamwera orthobunyavirus Gc glycoprotein head and stalk drives an infectious virion assembly pathway specific for the insect host
Source: PLoS Pathog. 2026 Jul 7;22(7):e1014374. doi: 10.1371/journal.ppat.1014374 (PMC13399505; doi:10.1371/journal.ppat.1014374)

SUPP Fig 16. Uncropped western blots from Supplemental Figure 8A; Immunoprecipitation of wildtype rBUNV-Gc-HA and Δ7 rBUNV-Gc-HA from A549 and C6/36 cells.

A – Panels 1-4 - Western cut and probed separately for HA and NP

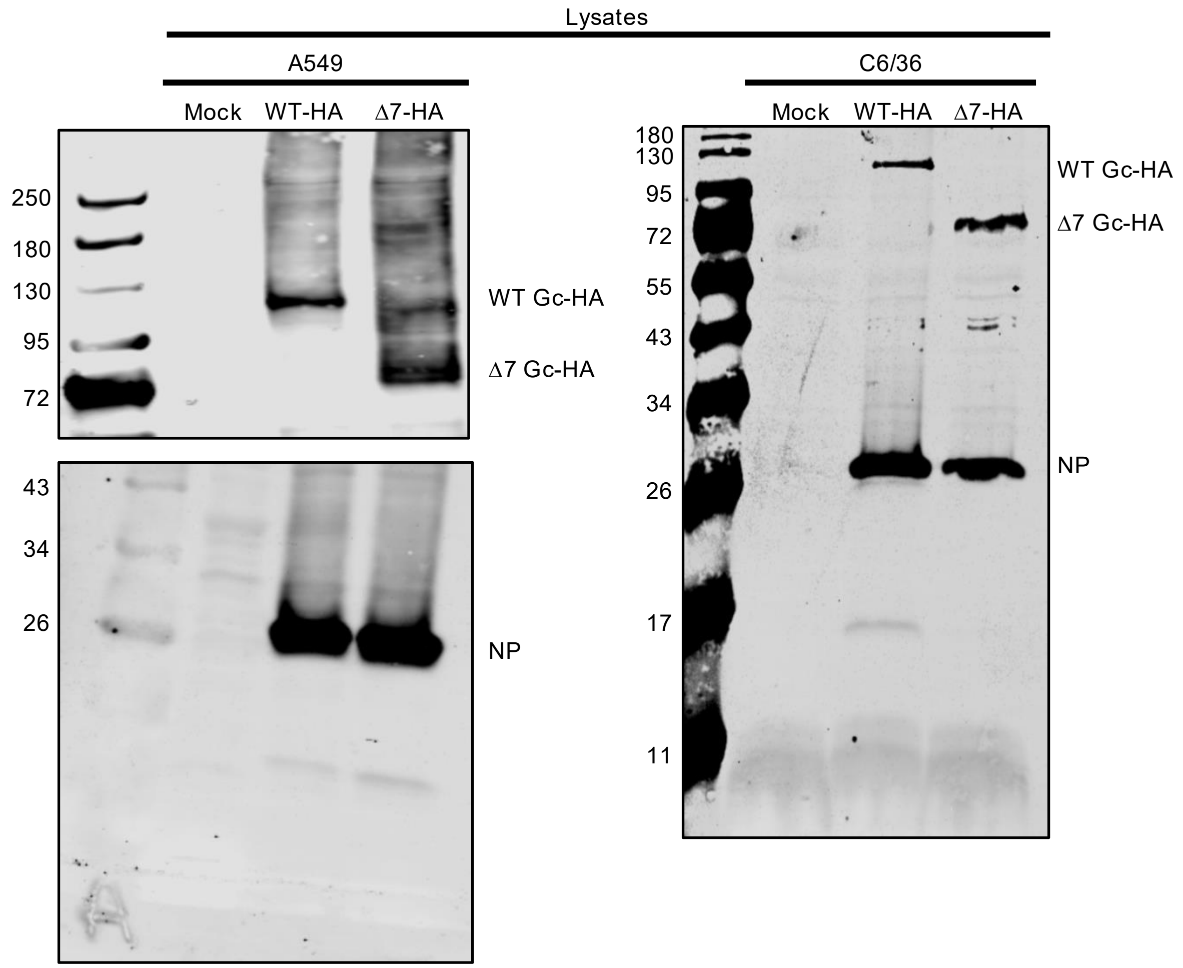

Supplement: S16 Fig — Uncropped western blots from lysates collected from A549 or C6/36 cells 24 hours post infection with either rBUNV-WT-Gc-HA (WT-HA) or rBUNV-∆7-Gc-HA (∆7-HA). The lysates (panels 1–4) were probed for expression of HA and NP, independently on western blots that were cut. (PDF) [file ppat.1014374.s016.pdf]
