# Supplementary material for: The Bunyamwera orthobunyavirus Gc glycoprotein head and stalk drives an infectious virion assembly pathway specific for the insect host
Source: PLoS Pathog. 2026 Jul 7;22(7):e1014374. doi: 10.1371/journal.ppat.1014374 (PMC13399505; doi:10.1371/journal.ppat.1014374)
Supplement: Attachment — Submitted filename: Response to reviewers 150526.docx [file ppat.1014374.s020.docx]

**Manuscript PPATHOGENS-D-25-02980: “The Bunyamwera orthobunyavirus Gc glycoprotein head and stalk drives an infectious virion assembly pathway that is specific for the insect host”**

Response to reviewers’ comments

Editors and reviewer’s comments in black text, our responses in blue text. Line numbers refer to the revised and marked up manuscript, unless stated otherwise.

**Editor comments:**

Thank you for your submission to PLoS Pathogens. All reviewers were in general agreement that this work addresses a compelling question in the field of arbovirology and the work presented is of high quality. However, major critiques of the submitted manuscript were noted and we encourage the authors to revise accordingly. Specifically, suggestions regarding choice of MOI for certain studies and confirming critical interactions by conventional IP and IFA should be considered.

We thank the editor for this comment and the reviewers for their positive comments. In the revised version of the manuscript, we have addressed the major critiques provided by the reviewers. We have included an additional experiment to address concerns regarding MOI (Supplemental Fig 3), as well as justification and explanation of changes to MOI in different experiments (lines 389; 403-404; 491; 778-779; 948-950). Furthermore, we have confirmed our mass spectrometry data with conventional pulldown assays by including another rep and repeating western blot and densitometry analysis, which improved statistical significance from a p value of 0.08 to 0.01 (Supplemental Fig 8). Finally, we have repeated immunofluorescence analysis including a membrane marker to permit quantification analysis of relocation of the Gc to the plasma membrane (Fig 5 and Supplemental Fig 7).

**Reviewer #1**

Major Issues:

(1) Many of the experiments were performed with an MOI of 5, a high MOI that is not clearly justified. What was the point of using such a high MOI? Why not a lower MOI that permit a comparison of the wt and ∆7 virus in the context of multiple rounds of assembly/exit/entry/replication? And did some experiments use an MOI of 1 used instead of 5? This is also not explained. The use of a high MOI needs to be justified and the implications of using this MOI vs. a lower MOI should be discussed.

We have included a justification for why we used MOI 5 in our initial experiments. Briefly, we wanted to simultaneously infect most cells and examine a single round of virus infection (lines 191-192). We have now also included a lower MOI experiment to investigate what happens in multiple rounds of infection (Supplemental Fig 3). This showed that rBUNV-∆7 titres are indistinguishable from WT also at low MOI in A549 cells. Crucially though, this was not seen in C6/36 cells, where formation of new infectious ∆7 viruses from C6/36 cells was restricted. We have also explained why in other experiments we used an MOI of 1 (lines 389; 403-404; 491; 778-780; 948-950), which was because we were trying to maintain a high MOI for examination of a single round of virus infection, but we were limited by the lower titre of mutant viruses.

(2) Related, line 177, relating how to interpret results at different timepoints: "The same three timepoints (6, 12 and 24 hpi) ... with 12 hpi reflecting virus released after just one round of infection, and 24 hpi reflecting virus released from multiple rounds of infection." What's the basis for saying this is what occurs following a high MOI infection?

We have rationalised our timepoints (lines 203-213) and included references of both high MOI and low MOI studies which show viruses start to be released at ~8 hpi, supporting our claim that 6 hpi reflects a time at which no new viruses has been generated, and that the 12 hpi timepoint represents one round of infection. We have included 24 hpi to allow infection of cells that were perhaps not infected during the initial infection.

(3) Is the ∆7 virus "assembly-deficient" (line 43) or is it just that the Gc is not in the right place for assembly to occur? This is partly a semantic point but it also relates to a distinction between the failure of a step upstream of assembly or a failure in assembly itself. The paper does not provide data to distinguish between these possibilities. The discussion explores these different explanations/hypotheses in more detail. But in other places, including in the title and abstract, the claim is that assembly itself is broken.

We appreciate this is a point of the paper that we have not been able to fully clarify experimentally, and we have made sure to address this in the discussion (lines 601-608). To further address the point raised here, we have softened claims where we said that ‘assembly’ is directly affected and we now refer to a deficiency in virion production (lines 48-49; 163; 169-170; 235; 564-565; 594-595; 627-628; 641-642) to appreciate that we are not sure which stage is affected, only that we know no new virions are generated. We have kept the title as is because we refer to the assembly pathway rather than specifically the stage of assembly.

(4) Similarly, is it that GP-NP interactions are decreased for the mutant, which cause a decrease in assembly, or is that virus particle production is less efficient, causing a decrease in detected GP-NP interactions (from GP-NP interactions in assembled particles). I also don't think the presented data distinguish between these possibilities.

We think that our data does distinguish between these possibilities. We agree that we cannot distinguish between whether the reduced Gc-NP interactions are the cause of disrupted assembly or the result of disrupted assembly and we have included this in our discussion (lines 605-608; 609-614). However, the reduction in Gc-NP interactions is seen from cell lysates (not supernatant), strongly suggesting that the decreased GP-NP interaction results in a decrease in assembly. Furthermore, we have included silver stain (Fig 4C) and western blot (Fig 4D) analysis of purified supernatant, which showed reduced NP and Gc bands for ∆7 (Fig 4E), alongside RT-qPCR data (Fig 4F) which showed a significant reduction in the ∆7 S genome. These methods suggest no new virions are generated. The decrease in detected Gc-NP interactions cannot be within assembled particles because we are not directly looking at Gc-NP interactions within virions and no new virions are generated from mutant infections.

(5) The paper does not provide sufficient evidence to support the assertion that "the complete Gc head/stalk assembly is maintained in nature due to its essential role in the insect host". This should be removed from the abstract, lines 47-48.

We have reworded this sentence in the abstract (lines 54-59) to make it clearer that this is a hypothesis we are proposing based on the data we have in the paper. Whilst we appreciate that we have not directly shown data for this, we provide sufficient evidence to propose this hypothesis, especially with a lack of identification of these mutants in insect populations in the literature.

(6) The experiment described in lines 289-299 involves mutation of putative/potential residues predicted to be involved in trimer formation but this prediction is based on structural prediction and was not confirmed by experimental assessment of trimer formation. Without this experimental confirmation, the conclusion that the "lack of head domain trimerisation was not the reason that rBUNV-∆7 viability is reduced in C6/36 cells" is not supported and must be removed. It is completely plausible that the ∆7 mutant is deficient in trimerisation.

We appreciate that we have not experimentally shown that these mutations disrupt the trimerisation of the Gc head domain, however, we think that it is still a useful experiment to have attempted and therefore we would prefer to keep this data in. We have tempered our conclusions to make it clear that the mutations we introduced, rather than a known disruption in trimerisation, are not the reason for the reduced rBUNV-∆7 viability in C6/36 cells (lines 407-409).

(7) Line 287: it would be more accurate to replace "is not the reason" with "cannot fully explain"

We have replaced “is not the reason for” with “does not fully explain” (line 395).

Minor Issues:

(8) Line 27-28: "...several important pathogens including the human-infecting Oropouche virus and animal-infecting Schmallenberg virus": humans and arthropods are animals too so animal-infecting here is not appropriate. Similar issue lines 52-53: "Arboviral OBV infections are persistent whereas in humans and animals OBV infection is associated with severe disease..."

We have replaced ‘animal’ with ‘ruminant’ to be more specific (lines 29; 35; 64; 69).

(9) Also line 53: "is associated with severe disease" should be replaced with something like "is sometimes associated with severe disease".

We have replaced “is associated with severe disease” in lines 64-65 with “can be associated with severe disease”.

(10) It would be helpful to include a cartoon depicting the location of naturally occurring deletions relative to the ∆7 deletion.

We have included a cartoon (Supplemental Fig 1) depicting the location of naturally occurring deletions present in the SBV isolates, alongside the location of BUNV-∆7 deletion and deletions found in isolates of passaged Maguari virus.

(11) The first introduction paragraph, lines 68-81, is lacking citations to support many factual claims. In contrast, the rest of the introduction is well cited.

We have added more citations to this paragraph (now lines 81-95).

(12) line 195: "confirm successful infection": infection should be replaced with ingestion.

We have replaced “infection” with “ingestion” (now line 239).

(13) The paragraph lines 445-456 describes host proteins identified in mass spec analyses but it seems like at least some of this belongs in results rather than discussion.

We appreciate the suggestion to describe some of the host proteins in the results section but because we have not further validated those findings with pulldown assays, we would prefer to keep these results in the discussion.

**Reviewer #2**

Major Issues:

(1) The description in Lines 26-35 of the Abstract is excessively redundant and should be condensed to 3-4 lines or fewer, with the focus on clarifying the identified differences between orthobunyaviruses derived from mammalian and insect hosts and the unknown molecular mechanisms underlying these discrepancies.

We have rephrased lines 27-38 of the abstract to focus more on the host-specific differences that we have presented in the paper and highlight that the underlying molecular mechanism is not understood.

(2) The authors focus on the core mechanism that "impaired viral assembly in insect cells is caused by reduced interaction between Gc and NP". It is recommended to merge Sentences 46–48 to enhance logical coherence. Meanwhile, please add an elaboration on the biological significance of this key mechanism.

We have reworded the abstract considering the reviewer’s suggestions. We have not merged sentences 53-59 though because the final sentence highlights the main biological significance of this key mechanism, which is our proposal of why the tripod is maintained in nature.

(3) Although BUNV is a representative strain of orthobunyaviruses (OBVs), the authors only demonstrated the importance of Gc integrity for BUNV assembly and release in mosquito cells. There is a lack of evidence to verify whether this conclusion is applicable to all OBV strains. Thus, the title is insufficiently specific: it is recommended to replace OBV with BUNV, unless experimental evidence supporting the universality of this mechanism across all OBVs is supplemented.

We appreciate that our studies have not been extended to other orthobunyaviruses and have adjusted the title and running title accordingly by replacing OBV with Bunyamwera virus.

(4) As an arbovirus, OBVs (including BUNV) raise critical transmissibility questions that require discussion (and empirical validation where necessary): whether Gc head/stalk -deficient virions produced in mammalian cells can be transmitted to mosquitoes; if transmissible, whether such Gc head/stalk -deficient viruses are capable of replication and amplification in mosquito cells; and whether the defective viruses can be re-transmitted to mammals thereafter. Relevant experimental data should be provided if available.

The data provided in the paper shows that that the Gc head/stalk deficient virions, produced in BHK mammalian cells for generation of the viral stock, are able to infect mosquito cells, express viral proteins and replicate, but unable to generate any new virions and therefore cannot progress infection within the insect cell culture. Because no new virions are generated, it cannot be tested whether the defective viruses can then be transmitted to mammalian cells.

(5) For the immunofluorescence images in Figures 5–6, please add annotations to indicate the enrichment of WT or Δ7-Gc on the plasma membrane, Golgi apparatus and other organelles, to confirm whether the Δ7 mutation perturbs the subcellular localization of Gc. In addition, quantitative analysis should be performed for all immunofluorescence images.

Whilst we appreciate annotations would be helpful, we could not confidently identify organelles without extensive immunostaining targeting the different organelles, plus there was no certainty that antibodies raised against mammalian organelle targets would detect those of insect origin. However, to address this suggestion, we repeated the immunofluorescence analysis including a stain for concanavalin A, prior to permeabilisation, to label the surface of the plasma membrane. Whilst this was not specific to the plasma membrane surface, we were able to use this signal to identify the edges in Fiji and create a mask that represented the surface. This allowed us to perform quantitative analysis of our images, determining that there was a significant increase in the presence of ∆7-Gc at the plasma membrane when compared to WT-Gc. These results are presented in an updated version of Figure 5 and in Supplemental Fig 7.

(6) The conclusion in Lines 408–410 is seemingly supported by mass spectrometry data, yet direct experimental evidence for the claim that "the interaction between ∆7-Gc-HA and NP is dramatically reduced in C6/36 cells" is absent. Supplementary validation assays (e.g., pull-down assay or surface plasmon resonance) are strongly recommended to substantiate this conclusion.

We have included western blot analysis of the pull-down assay performed prior to mass spectrometry analysis in Supplemental Figure 8. We have included a further replicate which has since made the reduction in NP precipitation by ∆7-Gc-HA from insect cells statistically significant (Supplemental Figure 8C). Therefore, we consider our conclusions to be sufficiently substantiated.

(7) The speculation in Lines 510–523 regarding the mechanism underlying the emergence of Gc deletion mutations in ruminant fetuses appears reasonable, but this inference is lacking relevant bibliographic references. Please supplement appropriate citations; if no literature is available, this speculative section should be revised with more cautious wording.

We have included appropriate citations to support our speculations (lines 670-690).

Minor Issues:

(8) The Introduction section is somewhat redundant and requires streamlining, especially the descriptions of general characteristics of OBVs. The revision should prioritize highlighting the specific roles and features of Gc across these OBV strains.

We thank you for your suggestion but we would prefer to keep the introduction section as it is currently. We believe that the introduction provides sufficient, relevant background on OBVs, keeping in mind researchers that are more interested in arthropods or arthropod viruses.

(9) Lines 137–153 should also be condensed to emphasize the major research findings, as well as the implications of these findings for viral assembly and even the viral transmission cycle between vector insects and mammals.

We have reworded the last paragraph of the introduction (lines 155-172) to emphasise the major research findings.

(10) In Lines 594–595, correct 1X TNE to 1× TNE.

We have replaced this with the standard multiplication symbol (lines 765-766).

(11) All instances of the uppercase letter X used to denote multiplication (e.g., Lines 632, 640, 654, 658, 670 and so on) should be revised to the standard multiplication symbol ×; a full-text check and correction are required for this inconsistency.

We have replaced all instances with the standard multiplication symbol.

(12) Some experimental descriptions (e.g., virus purification, electron microscopy sample preparation) are overly detailed and verbose. Consider relocating these details to Supplementary Materials or simplifying the textual descriptions in the main manuscript.

We have reduced the detail in these experimental descriptions (lines 819-837; 840-863).

(13) While the analytical methodology for mass spectrometry is comprehensively described, certain technical details (e.g., database version, search parameters) may be moved to the Supplementary Methods/Appendix for brevity of the main text.

We have reduced the detail in the experimental descriptions of the mass spectrometry (lines 964-1074).

**Reviewer #3**

Major Issues:

(1) Figure 1. To assess the multiplication characteristics of rBUNV WT and rBUNV-Δ7 in A549, BHK-21, and C6/36 cells, the authors performed time-course experiments at a MOI = 5. Based on these data, they conclude that the viruses were able to enter and perform gene expression in all three cell lines in an equivalent manner (lines 174-175). However, high-MOI conditions largely restrict the analysis to a single replication cycle. Inclusion of low-MOI experiments (e.g., MOI = 0.01) would allow evaluation of multi-cycle growth and provide a more robust comparison of viral replication features. Consistent with predominantly single-cycle infection, viral titers increase by less than one log between 12 and 24 h post-infection in mammalian cells, and infectious rBUNV-Δ7 titers fail to increase in C6/36 supernatants (Fig. 1C). This phenotype is more consistent with defects in early infection steps (e.g., viral entry or M-segment polyprotein expression/processing) than with a selective defect in virus assembly.

We have included low MOI experiments performed at MOI 0.01 and harvested at 24h and 48h to give sufficient time for generation of new viral particles (Supplemental Fig 3). We detected a similar phenotype to the one seen in the high MOI experiment, whereby in A549 cells, BUNV ∆7 reached similar (not significantly different) titres to BUNV WT by 48 h, but at this timepoint in C6/36 cells titre is 5-log lower than BUNV WT. This indicates no new BUNV-∆7 viruses are generated and BUNV-∆7 is incapable of spread throughout insect cells.

(2) Similarly, the data presented in Figure 3 do not exclude defects in viral entry or M-segment polyprotein synthesis in insect cells, as both intracellular and extracellular viral titers remain close to inoculum levels. Accordingly, the conclusions drawn from these experiments (shown in Figure 1 and 3) should be appropriately moderated.

In Fig 1, we have shown NP expression is similar in C6/36 cells for WT and ∆7, which confirms that entry and initial protein expression are not affected by the deletion. We now present the results confirming that Gc-HA is expressed correctly earlier in the paper as a means to prove that Gc expression and processing is also unaffected by the deletion (lines 259-299; now Fig 3). Therefore we can be confident in our conclusions for the previous Fig 3 (now Fig 4).

(3) In my view, evidence for viral entry and M-segment synthesis/processing of the Δ7 virus in C6/36 cells is provided in Figure 4, where a clear and progressive increase in both HA-Gc and NP levels is observed over time.

We have rearranged the results section to introduce the results confirming that Gc-HA is expressed correctly earlier in the paper as a means to prove that Gc expression and processing is unaffected by the deletion (lines 259-299; Fig 3).

(4) The in vivo mosquito experiment is informative, but does not directly address a defect in viral assembly and does not exclude an entry defect. Demonstration of early viral RNA (e.g., by RT-PCR) would be required to confirm successful infection. In the absence of such evidence, the corresponding conclusions should be tempered. Moreover, based on the data presented, the possibility of impaired viral entry cannot be disregarded in the context of whole-organism infection (mosquitoes or mammals), and these limitations should be more explicitly discussed in the discussion section.

Unfortunately, we do not have mosquitos harvested at an early timepoint with which to perform RT-PCR and demonstrate successful infection. We have altered our conclusions (lines 250-254) and discussed the possibility of *in vivo* experiments in the discussion, as well as discussing the need for investigation within a mammalian host (lines 556-565).

(5) Figure 5. While the NP staining pattern in rBUNV-WT-Gc-HA and rBUNV-Δ7-Gc-HA infected cells is similar at equivalent time points, at 24 h post-infection NP clearly exhibits a more peripheral distribution, potentially proximal to the plasma membrane, which is not evident at 18 h (lines 337-338). Please revise.

The new immunofluorescence data presented in Fig 5 and Supplemental Fig 7 allowed us to colocalise NP (and Gc) to the plasma membrane. The data we present showed that while ∆7-Gc-HA localised more to the plasma membrane that WT-Gc-HA, this was not seen for ∆7-NP.

(6) Quantitative analysis of NP localization (e.g., radial intensity profiling), together with co-staining for plasma membrane or cortical actin markers, would help determine whether this redistribution reflects a biologically meaningful change. In addition, beyond widefield microscopy, a more comprehensive analysis of the degree of overlap between NP and Gc-HA, using confocal z-stack imaging across the entire cell under different conditions, would substantially strengthen interpretation of these data. This is particularly relevant given that the reduced interaction observed in Figure 7 may, at least in part, reflect decreased spatial overlap between NP and Δ7-Gc-HA.

We have repeated our immunofluorescence analysis including a stain for concanavalin A prior to permeabilisation, to label only the surface of the plasma membrane. Whilst this was not specific to the plasma membrane surface, we were able to use this signal to identify the edges in Fiji and create a mask that represents the surface. This allowed us to perform quantitative analysis of our images (Fig 5C and Supplemental Fig 7C), determining that there was a biologically significant increase in the presence of ∆7-Gc at the plasma membrane when compared to WT-Gc. Furthermore, we collected this data on a confocal microscope to improve the analysis. Due to the high expression of intracellular puncta of Gc for both WT and ∆7, which spatially overlapped with the cytoplasmic diffuse NP expression, we were unable to perform biologically relevant quantitative analysis of Gc-HA overlap with NP.

(7) Figure S4. The co-IP experiment lacks input controls. Differences in the amount of co-IP NP may therefore reflect differences in protein abundance rather than changes in interaction efficiency.

We have included lysate input controls for the co-IP experiment to show that the reduction in NP precipitation from rBUNV-∆7-infected C6/36 cells is not due to a lower protein abundance in the input lysates (now Supplemental Fig 8A).

Minor Issues:

(8) In Line 137, the authors state: “Here, we wanted to further investigate the role of the OBV Gc head and stalk domains, to better understand why they are apparently dispensable for multiplication in the mammalian host, yet are maintained in nature.” As the experiments were performed exclusively in mammalian cell lines rather than in a mammalian host model, the wording should be revised accordingly (e.g., replacing “mammalian host” with “mammalian cells”).

We have replaced mammalian host with mammalian cells (line 157).

(9) Lines 156-157. The statement that rBUNV-Δ7 exhibits “no fitness loss” in mammalian cells is too broad, as viral fitness encompasses parameters not directly assessed here. Replacing this wording with a more specific description of the measured phenotype (e.g., multiplication or virus production) would be more appropriate.

We have changed the title of this section to have a more specific reflection of the data. The new section title is “rBUNV-Δ7 produces infectious virus in mammalian cells but not in insect cells” (line 175-176).

(10) I suggest replacing “Gc-HA” with “HA-Gc” throughout the manuscript (text and figures) to accurately reflect the N-terminal position of the HA tag.

We appreciate this suggestion to reflect the N-terminal location of the HA tag, but because the HA tag is derived from another viral glycoprotein, we feel that placing HA before Gc could imply that the HA glycoprotein is tagged with Gc. Furthermore, we feel that when discussing WT versus ∆7 Gc, the nomenclature should start with either WT or ∆7 but to follow this with HA-Gc instead of Gc-HA gives the impression that the WT or mutant affects HA not Gc.

(11) The title of Figure S4 (both in the legend and in the figure file) does not correspond to the data shown in the figure and should be corrected.

We would like to thank the reviewer for identifying this mistake and we have corrected the title to “Immunoprecipitation of wildtype rBUNV-Gc-HA and ∆7 rBUNV-Gc-HA from A549 and C6/36 cells.” (now Supplemental Fig 8).

**Reviewer #4**

Major Issues:

(1) Figure 1: The authors should confirm the defect of infectious titer by another methods than plaque assay (by IF for example). Indeed, it would be interesting to check that the ∆7 did not lose its capacity to perform plaques in infected cells.

As per suggestions 3 and 4 by this reviewer (points 3 & 4), we have now quantified secreted genome, alongside silver stain blot analysis of purified supernatant from infected C6/36 cells infected with BUNV ∆7, to confirm the lack of infectious secreted viruses.

(2) Figure 1: The authors should repeat the experiment with a lower MOI to confirm that the effect of the delta7 is not linked to a defect of spread that could be mitigated by the MOI used.

We now include experiments performed at MOI 0.01 and harvested at 24h and 48h to give sufficient time for generation of new viral particles (Supplemental Fig 3). We detect a similar phenotype to the high MOI one, whereby in A549 cells, BUNV ∆7 reaches similar (not significantly different) titres, but in C6/36 cells titre is 5-log lower than BUNV WT. This indicates no new BUNV-∆7 viruses were generated and BUNV-∆7 was incapable of spread throughout the cells.

(3) Figure 3C: The authors should add a “Mock” sample obtained from uninfected C6/36 to confirm the identification of the bands. In addition, the authors should perform a WB for quantification of NP as well as Gc (using, if necessary, HA tag antibodies since they generated HA-tagged version of Gc).

We have performed a purification of supernatant collected from mock-infected C6/36 cells to assist identification of viral protein bands by silver stain analysis (Fig 4C). Furthermore, we performed purification of HA-tagged rBUNV to allow western blot analysis of Gc and NP bands (Fig 4D) and subsequent densitometry quantification (*n=2*) (Fig 4E), which shows a decrease in both Gc and NP expression in purified BUNV-∆7 pellets.

(4) Figure 3: To complement this figure, the authors should check the level of secreted genome (at least for one segment) to confirm the lack of secretion of viral particles.

We performed RT-qPCR (Fig 4F) to quantify BUNV S genome copies present in the supernatant of C6/36 cells either mock-infected or infected with rBUNV-WT-Gc-HA or rBUNV-∆7-Gc-HA. This confirmed that we were able to extract ~10^7^ BUNV S copies/mL from WT-infected cells but were unable to detect any BUNV S copies from ∆7-infected cells, which meant any ∆7 S RNA was below the limit of detection for our assay (which was 2.5x10^4^ copies/mL). This tallies with previous titre data and confirms that there is a lack of secreted non-infectious virus particles in rBUNV-∆7 C6/36-infected cells.

(5) Line 240: EM data of supernatants of BUNV-WT vs. rBUNV-Δ7-infected C6/36 cells should be shown.

We were reluctant to show EM data of the lack of rBUNV-∆7 viruses purified from C6/36 cells due to the presence of endogenous viruses purified from the insect cell culture, which is something that has been described before. Whilst these viruses were clearly different from rBUNV-WT, it was distracting to include the data. Therefore, we have removed any mentions of this data and instead proved the lack of virus-like particles by silver stain analysis (Fig 4C), western blot analysis (Fig 4D) and RT-qPCR analysis (Fig 4F).

(6) Figure 5: The authors should perform the same IF on mammalian cells to check the lack of altered distribution of Gc for the mutant. They should also better assess their hypotheses that Gc/NP are localized at Golgi vs. plasma membrane (for Gc, using specific markers of the above.

We repeated our immunofluorescence analysis including a stain for concanavalin A, prior to permeabilization to label the surface of the plasma membrane (Fig 5 and Supplemental Fig 7). We then used this signal to identify the edges in Fiji and create a mask that represented the surface. This allowed us to perform quantitative analysis of our images (Fig 5C and Supplemental Fig 7C), determining that there was a biologically significant increase in the presence of ∆7-Gc at the plasma membrane when compared to WT-Gc. There did not appear to be a visual difference in ∆7-Gc localisation to cytoplasmic puncta, which we hypothesise represent the Golgi, plus there was no certainty that antibodies raised against mammalian organelle targets would detect those of insect origin. Therefore, we decided not to attempt quantification analysis of the Golgi.

(7) Figures 5 and 6: Please provide quantifications the data with statistical analyses to consolidate these findings (along with Golgi/plasma membrane markers).

We have collected new data on a confocal microscope and performed quantitative analysis of Gc and NP co-occurrence with a mask representing the plasma membrane (Fig 5C and Supplemental Fig 7C). This allowed us to perform statistical analyses which showed a statistically significant increase in the presence of ∆7-Gc-HA at the plasma membrane of C6/36 cells.

(8) Owing to pretty intense filopodia Gc staining of delta7 BUNV in C6/36 cells, the authors should address the possibility that the reduced cell-free transmission of this mutant virus could be compensated by cell-cell transmission. Thus, cell-cell transmission assays should be performed to address this possibility.

Given that because there is no accumulation of intracellular infectious particles (Supplemental Fig 5), we believe cell-to-cell transmission is unlikely to be happening. Additionally, it would be similarly impaired by the reduction in NP-Gc interaction.

(9) SUPP Figure 4 and Figure 7. While the data of TMT-MS will be important for further investigations of host factors that regulate assembly in insect cells, they suggest that the ability of Δ7-Gc-HA to interact with NP is reduced in C6/36 cells. Yet, the authors failed to validate this result by classical coIP assays (SUPP Figure 4), although the results show a clear tendency of reduced Gc/NP coIP for the Δ7 mutant in insect cells. These assays should therefore be repeated, perhaps optimized to avoid the potential “presence of contaminating antibody bands at the same molecular weight as NP” (which is not obvious to me).

We have performed the coIP assay for another rep (n=4) (Supplemental Fig 8A-B) and repeated the densitometry analysis (Supplemental Fig 8C), which resulted in a statistically significant reduction in NP precipitated by ∆7-Gc in insect cells. We have included lysate input controls (Supplemental Fig 8A) for the co-IP experiment to show that the reduction in NP precipitation from rBUNV-∆7-infected C6/36 cells is not due to a lower protein abundance in the input lysates.

Minor Issues:

(10) The authors have put a lot of efforts to demonstrate the decrease association between Gc and NP (probably as RNP), but this might just be the consequence of the impaired localization of Gc, and the mechanism regarding this remains not understood. This should therefore be highlighted in the Discussion.

We appreciate this input and have addressed this further in the Discussion (lines 609-614).
