# Supplementary material for: The Bunyamwera orthobunyavirus Gc glycoprotein head and stalk drives an infectious virion assembly pathway specific for the insect host
Source: PLoS Pathog. 2026 Jul 7;22(7):e1014374. doi: 10.1371/journal.ppat.1014374 (PMC13399505; doi:10.1371/journal.ppat.1014374)
Supplement: Peer review history

# Peer review: Decision Letter 0

**DOI:** 10.1371/journal.ppat.1014374.r001

**Authors:** Matthias Schnell, Alison Kell

12 Jan 2026

-->PPATHOGENS-D-25-02980

The orthobunyavirus Gc glycoprotein head and stalk drives an infectious virion assembly pathway specific for the insect host

PLOS Pathogens

Dear Dr. Barr,

Thank you for submitting your manuscript to PLOS Pathogens. After careful consideration, we feel that it has merit but does not fully meet PLOS Pathogens's publication criteria as it currently stands. Therefore, we invite you to submit a revised version of the manuscript that addresses the points raised during the review process.

Please submit your revised manuscript by Mar 13 2026 11:59PM. If you will need more time than this to complete your revisions, please reply to this message or contact the journal office at plospathogens@plos.org. When you're ready to submit your revision, log on to https://www.editorialmanager.com/ppathogens/ and select the 'Submissions Needing Revision' folder to locate your manuscript file.

Please include the following items when submitting your revised manuscript:

* A letter that responds to each point raised by the editor and reviewer(s). You should upload this letter as a separate file labeled 'Response to Reviewers'. This file does not need to include responses to any formatting updates and technical items listed in the 'Journal Requirements' section below.

* A marked-up copy of your manuscript that highlights changes made to the original version. You should upload this as a separate file labeled 'Revised Manuscript with Track Changes'.

* An unmarked version of your revised paper without tracked changes. You should upload this as a separate file labeled 'Manuscript'.

If you would like to make changes to your financial disclosure, competing interests statement, or data availability statement, please make these updates within the submission form at the time of resubmission. Guidelines for resubmitting your figure files are available below the reviewer comments at the end of this letter.

We look forward to receiving your revised manuscript.

Kind regards,

Alison M Kell

Academic Editor

PLOS Pathogens

Matthias Schnell

Section Editor

PLOS Pathogens

Sumita Bhaduri-McIntosh

Editor-in-Chief

PLOS Pathogens

orcid.org/0000-0003-2946-9497

Michael Malim

Editor-in-Chief

PLOS Pathogens

orcid.org/0000-0002-7699-2064

**Additional Editor Comments:**

Thank you for your submission to PLoS Pathogens. All reviewers were in general agreement that this work addresses a compelling question in the field of arbovirology and the work presented is of high quality. However, major critiques of the submitted manuscript were noted and we encourage the authors to revise accordingly. Specifically, suggestions regarding choice of MOI for certain studies and confirming critical interactions by conventional IP and IFA should be considered.

**Journal Requirements:**

If the reviewer comments include a recommendation to cite specific previously published works, please review and evaluate these publications to determine whether they are relevant and should be cited. There is no requirement to cite these works unless the editor has indicated otherwise.

1) Please ensure that the CRediT author contributions listed for every co-author are completed accurately and in full.

At this stage, the following Authors/Authors require contributions: Amelia Briony Shaw. Please ensure that the full contributions of each author are acknowledged in the "Add/Edit/Remove Authors" section of our submission form.

The list of CRediT author contributions may be found here: https://journals.plos.org/plospathogens/s/authorship#loc-author-contributions

2) We noticed that you used the phrase 'data not shown' in the manuscript. We do not allow these references, as the PLOS data access policy requires that all data be either published with the manuscript or made available in a publicly accessible database. Please amend the supplementary material to include the referenced data or remove the references.

3) We do not publish any copyright or trademark symbols that usually accompany proprietary names, eg ©,  ®, or TM  (e.g. next to drug or reagent names). Therefore please remove all instances of trademark/copyright symbols throughout the text, including:

- TM on pages: 24, 25, and 26.

4) Please upload all main figures as separate Figure files in .tif or .eps format. For more information about how to convert and format your figure files please see our guidelines:

https://journals.plos.org/plospathogens/s/figures

5) We have noticed that you have uploaded Supporting Information files, but you have not included a list of legends. Please add a full list of legends for your Supporting Information files after the references list.

6) Please amend your detailed Financial Disclosure statement. This is published with the article. It must therefore be completed in full sentences and contain the exact wording you wish to be published.

1) State what role the funders took in the study. If the funders had no role in your study, please state: "The funders had no role in study design, data collection and analysis, decision to publish, or preparation of the manuscript."

2) If any authors received a salary from any of your funders, please state which authors and which funders..

If you did not receive any funding for this study, please simply state: u201cThe authors received no specific funding for this work.u201d

**Reviewers' Comments:**

Reviewer's Responses to Questions

**Part I - Summary**

Please use this section to discuss strengths/weaknesses of study, novelty/significance, general execution and scholarship.

Reviewer #1: Shaw and colleagues investigate the phenotype of a large deletion in the bunyamwera virus glycoprotein. This deletion, called ∆7, removes about a quarter of the glycoprotein. Orthobunyavirus GP deletions have been recovered from mammalian samples in nature but not from arthropod samples. This large deletion has surprisingly little impact in mammalian cells but results in decreased titer from mosquito cells and failure of infection in mosquitoes. The authors explore this species-specific difference and find that GP∆7 levels and trafficking are different than wild type GP.

This paper is well done and well written and contributes to a better understanding of species-specific differences in arbovirus molecular virology. The connection with naturally occurring deletions is compelling. The paper could be improved in several ways, in particular by revising conclusions that are not sufficiently supported by the presented data.

Reviewer #2: The authors systematically compare the replication, assembly, and release of wild-type BUNV with the Gc head/stalk deletion mutant (Δ7) in mammalian versus insect cells. Their work clearly demonstrates the essential and host-specific role of the Gc head/stalk domains for virion assembly in the insect host. The experimental approach is diverse and robust, including viral growth kinetics, mosquito infection studies, co-immunoprecipitation coupled with mass spectrometry, and immunofluorescence localization. The data strongly support the conclusions. Although the story is solidly grounded, several suggestions still need to be further strengthened before publication.

Reviewer #3: Although orthobunyavirus entry is generally thought to be mediated by interactions between viral envelope glycoproteins and host cell receptors, a previous study demonstrated that, in Bunyamwera virus (BUNV), large portions of the Gc head and stalk domains are dispensable for viral multiplication in mammalian cells. Notably, one variant (Δ7) displayed growth kinetics comparable to wild-type BUNV despite deletion of the entire head domain and most of the stalk region. In the present study, by Shaw et al, the authors seek to understand why these regions are dispensable for replication in mammalian cells, yet conserved in nature. In this regard, they confirmed the previous observations in mammalian cells, but show that BUNV replication in insect cells and mosquitoes requires an intact Gc head/stalk assembly. They also provide evidence that these domains contribute to virus assembly in insect cells, potentially by mediating interactions between Gc and the nucleoprotein (NP). Finally, they perform co-IP paired with and multiplex mass spectrometry to map viral and host proteins that differentially interact with wt and Δ7-Gc in both insect and mammalian cells.

The manuscript provides novel important information, is clearly written, and presents high-quality data that, for the most part, support the authors’ conclusions.

I have only a few comments and suggestions.

Reviewer #4: Fully infectious variants of several members of the Orthobunyavirus genus that lack a large part of the Gc head/stalk domains have been described a long time ago from mammalian cells and/or hosts; yet, such mutants have never been isolated from arthropod hosts although such virus cycle between these two host types in nature. Here, the authors sought to address the molecular bases for the functional requirements of Gc that seem different across mammalian vs. mosquito cells. Using a genetically engineered variant of Bunyamwera virus (BUNV) that lacks the head/stalk domains, termed delta-7 (Δ7), and that is fully infectious in mammalian cells, they show how the head and stalk Gc domain drive a distinct pathway of assembly of infectious virion that is specific for the insect host. They specifically observed redistribution of Δ7-Gc to the plasma membrane and a significant reduction in the ability of the head/stalk truncated Gc of rBUNV-Δ7 to interact with NP in insect cells, which was not seen in mammalian cells, indicating that Gc plays a role in RNP recruitment and that its deletion blocks virion assembly in insect cells.

Overall, this study is particularly interesting since, based on the notion that arboviruses need to cope with fairly distinct environments to produce infectious particles, it addresses assembly pathways that seem distinct in either host. Furthermore, they uncovered some involved viral determinant and, though it will deserve further studies, identify potential host factors in insect cells that could contribute to explain such marked differences in viral assembly.

The experimental work is well performed and we make some suggestions to improve the manuscript.

**********

**Part II – Major Issues: Key Experiments Required for Acceptance**

Please use this section to detail the key new experiments or modifications of existing experiments that should be absolutely required to validate study conclusions.

Generally, there should be no more than 3 such required experiments or major modifications for a "Major Revision" recommendation. If more than 3 experiments are necessary to validate the study conclusions, then you are encouraged to recommend "Reject".

Reviewer #1: (*) Many of the experiments were performed with an MOI of 5, a high MOI that is not clearly justified. What was the point of using such a high MOI? Why not a lower MOI that permit a comparison of the wt and ∆7 virus in the context of multiple rounds of assembly/exit/entry/replication? And did some experiments use an MOI of 1 used instead of 5? This is also not explained. The use of a high MOI needs to be justified and the implications of using this MOI vs. a lower MOI should be discussed.

Related, line 177, relating how to interpret results at different timepoints: "The same three timepoints (6, 12 and 24 hpi) ... with 12 hpi reflecting virus released after just one round o infection, and 24 hpi reflecting virus released from multiple rounds of infection." What's the basis for saying this is what occurs following a high MOI infection?

(*) Is the ∆7 virus "assembly-deficient" (line 43) or is it just that the Gc is not in the right place for assembly to occur? This is partly a semantic point but it also relates to a distinction between the failure of a step upstream of asesembly or a failure in assembly itself. The paper does not provides data to distinguish between these possibilities. The discussion explores these different explanations/hypotheses in more detail. But in other places, including in the title and abstract, the claim is that assembly itself is broken.

(*) Similarly, is it that GP-NP interactions are decreased for the mutant, which cause a decrease in assembly, or is that virus particle production is less efficient, causing a decrease in detected GP-NP interactions (from GP-NP interactions in assembled particles). I also don't think the presented data distinguish between these possibilities.

(*) The paper does not provide sufficient evidence to support the assertion that "the complete Gc head/stalk assembly is maintained in nature due to its essential role in the insect host". This should be removed from the abstract, lines 47-48.

(*) The experiment described in lines 289-299 involves mutation of putative/potential residues predicted to be involved in trimer formation but this prediction is based on structural prediction and was not confirmed by experimental assessment of trimer formation. Without this experimental confirmation, the conclusion that the "lack of head domain trimerisation was not the reason that rBUNV-∆7 viability is reduced in C6/36 cells" is not supported and must be removed. It is completely plausible that the ∆7 mutant is deficient in trimerisation.

(*) Line 287: it would be more accurate to replace "is not the reason" with "cannot fully explain"

Reviewer #2: 1.The description in Lines 26-35 of the Abstract is excessively redundant and should be condensed to 3-4 lines or fewer, with the focus on clarifying the identified differences between orthobunyaviruses derived from mammalian and insect hosts and the unknown molecular mechanisms underlying these discrepancies.

2.The authors focus on the core mechanism that "impaired viral assembly in insect cells is caused by reduced interaction between Gc and NP". It is recommended to merge Sentences 46–48 to enhance logical coherence. Meanwhile, please add an elaboration on the biological significance of this key mechanism.

3.Although BUNV is a representative strain of orthobunyaviruses (OBVs), the authors only demonstrated the importance of Gc integrity for BUNV assembly and release in mosquito cells. There is a lack of evidence to verify whether this conclusion is applicable to all OBV strains. Thus, the title is insufficiently specific: it is recommended to replace OBV with BUNV, unless experimental evidence supporting the universality of this mechanism across all OBVs is supplemented.

4.As an arbovirus, OBVs (including BUNV) raise critical transmissibility questions that require discussion (and empirical validation where necessary): whether Gc head/stalk -deficient virions produced in mammalian cells can be transmitted to mosquitoes; if transmissible, whether such Gc head/stalk -deficient viruses are capable of replication and amplification in mosquito cells; and whether the defective viruses can be re-transmitted to mammals thereafter. Relevant experimental data should be provided if available.

5.For the immunofluorescence images in Figures 5–6, please add annotations to indicate the enrichment of WT or Δ7-Gc on the plasma membrane, Golgi apparatus and other organelles, to confirm whether the Δ7 mutation perturbs the subcellular localization of Gc. In addition, quantitative analysis should be performed for all immunofluorescence images.

6.The conclusion in Lines 408–410 is seemingly supported by mass spectrometry data, yet direct experimental evidence for the claim that "the interaction between ∆7-Gc-HA and NP is dramatically reduced in C6/36 cells" is absent. Supplementary validation assays (e.g., pull-down assay or surface plasmon resonance) are strongly recommended to substantiate this conclusion.

7.The speculation in Lines 510–523 regarding the mechanism underlying the emergence of Gc deletion mutations in ruminant fetuses appears reasonable, but this inference is lacking relevant bibliographic references. Please supplement appropriate citations; if no literature is available, this speculative section should be revised with more cautious wording.

Reviewer #3: Figure 1. To assess the multiplication characteristics of rBUNV WT and rBUNV-Δ7 in A549, BHK-21, and C6/36 cells, the authors performed time-course experiments at a MOI = 5. Based on these data, they conclude that the viruses were able to enter and perform gene expression in all three cell lines in an equivalent manner (lines 174-175). However, high-MOI conditions largely restrict the analysis to a single replication cycle. Inclusion of low-MOI experiments (e.g., MOI = 0.01) would allow evaluation of multi-cycle growth and provide a more robust comparison of viral replication features. Consistent with predominantly single-cycle infection, viral titers increase by less than one log between 12 and 24 h post-infection in mammalian cells, and infectious rBUNV-Δ7 titers fail to increase in C6/36 supernatants (Fig. 1C). This phenotype is more consistent with defects in early infection steps (e.g., viral entry or M-segment polyprotein expression/processing) than with a selective defect in virus assembly.

Similarly, the data presented in Figure 3 do not exclude defects in viral entry or M-segment polyprotein synthesis in insect cells, as both intracellular and extracellular viral titers remain close to inoculum levels. Accordingly, the conclusions drawn from these experiments (shown in Figure 1 and 3) should be appropriately moderated.

In my view, evidence for viral entry and M-segment synthesis/processing of the Δ7 virus in C6/36 cells is provided in Figure 4, where a clear and progressive increase in both HA-Gc and NP levels is observed over time.

The in vivo mosquito experiment is informative, but does not directly address a defect in viral assembly and does not exclude an entry defect. Demonstration of early viral RNA (e.g., by RT-PCR) would be required to confirm successful infection. In the absence of such evidence, the corresponding conclusions should be tempered. Moreover, based on the data presented, the possibility of impaired viral entry cannot be disregarded in the context of whole-organism infection (mosquitoes or mammals), and these limitations should be more explicitly discussed in the discussion section.

Figure 5. While the NP staining pattern in rBUNV-WT-Gc-HA and rBUNV-Δ7-Gc-HA infected cells is similar at equivalent time points, at 24 h post-infection NP clearly exhibits a more peripheral distribution, potentially proximal to the plasma membrane, which is not evident at 18 h (lines 337-338). Please revise.

Quantitative analysis of NP localization (e.g., radial intensity profiling), together with co-staining for plasma membrane or cortical actin markers, would help determine whether this redistribution reflects a biologically meaningful change. In addition, beyond widefield microscopy, a more comprehensive analysis of the degree of overlap between NP and Gc-HA, using confocal z-stack imaging across the entire cell under different conditions, would substantially strengthen interpretation of these data. This is particularly relevant given that the reduced interaction observed in Figure 7 may, at least in part, reflect decreased spatial overlap between NP and Δ7-Gc-HA.

Figure S4. The co-IP experiment lacks input controls. Differences in the amount of co-IP NP may therefore reflect differences in protein abundance rather than changes in interaction efficiency.

Reviewer #4: - Figure 1: The authors should confirm the defect of infectious titer by another methods than plaque assay (by IF for example). Indeed, it would be interesting to check that the 7 did not lose its capacity to perform plaques in infected cells.

- Figure 1: The authors should repeat the experiment with a lower MOI to confirm that the effect of the delta7 is not linked to a defect of spread that could be mitigated by the MOI used.

- Figure 3C: The authors should add a “Mock” sample obtained from uninfected C6/36 to confirm the identification of the bands. In addition, the authors should perform a WB for quantification of NP as well as Gc (using, if necessary, HA tag antibodies since they generated HA-tagged version of Gc).

- Figure 3: To complement this figure, the authors should check the level of secreted genome (at least for one segment) to confirm the lack of secretion of viral particles.

- Line 240: EM data of supernatants of BUNV-WT vs. rBUNV-Δ7-infected C6/36 cells should be shown.

- Figure 5: The authors should perform the same IF on mammalian cells to check the lack of altered distribution of Gc for the mutant. They should also better assess their hypotheses that Gc/NP are localized at Golgi vs. plasma membrane (for Gc), using specific markers of the above.

- Figures 5 and 6: Please provide quantifications the data with statistical analyses to consolidate these findings (along with Golgi/plasma membrane markers).

- Owing to pretty intense filopodia Gc staining of delta7 BUNV in C6/36 cells, the authors should address the possibility that the reduced cell-free transmission of this mutant virus could be compensated by cell-cell transmission. Thus, cell-cell transmission assays should be performed to address this possibility.

- SUPP Figure 4 and Figure 7. While the data of TMT-MS will be important for further investigations of host factors that regulate assembly in insect cells, they suggest that the ability of Δ7-Gc-HA to interact with NP is reduced in C6/36 cells. Yet, the authors failed to validate this result by classical coIP assays (SUPP Figure 4), although the results show a clear tendency of reduced Gc/NP coIP for the Δ7 mutant in insect cells. These assays should therefore be repeated, perhaps optimized to avoid the potential “presence of contaminating antibody bands at the same molecular weight as NP” (which is not obvious to me).

**********

**Part III – Minor Issues: Editorial and Data Presentation Modifications**

Please use this section for editorial suggestions as well as relatively minor modifications of existing data that would enhance clarity.

Reviewer #1: (*) Line 27-28: "...several important pathogens including the human-infecting Oropouche virus and animal-infecting Schmallenberg virus": humans and arthropods are animals too so animal-infecting here is not appropriate. Similar issue lines 52-53: "Arboviral OBV infections are persistent whereas in humans and animals OBV infection is associated with severe disease..."

Also line 53: "is associated with severe disease" should be replaced with something like "is sometimes associated with severe disease".

(*) It would be helpful to include a cartoon depicting the location of naturally occurring deletions relative to the ∆7 deletion.

(*) The first introduction paragraph, lines 68-81, is lacking citations to support many factual claims. In contrast, the rest of the introduction is well cited.

(*) line 195: "confirm successful infection": infection should be replaced with ingestion.

(*) The paragraph lines 445-456 describes host proteins identified in mass spec analyses but it seems like at least some of this belongs in results rather than discussion.

Reviewer #2: 1.The Introduction section is somewhat redundant and requires streamlining, especially the descriptions of general characteristics of OBVs. The revision should prioritize highlighting the specific roles and features of Gc across these OBV strains.

2.Lines 137–153 should also be condensed to emphasize the major research findings, as well as the implications of these findings for viral assembly and even the viral transmission cycle between vector insects and mammals.

3.In Lines 594–595, correct 1X TNE to 1× TNE.

4.All instances of the uppercase letter X used to denote multiplication (e.g., Lines 632, 640, 654, 658, 670 and so on) should be revised to the standard multiplication symbol ×; a full-text check and correction are required for this inconsistency.

5.Some experimental descriptions (e.g., virus purification, electron microscopy sample preparation) are overly detailed and verbose. Consider relocating these details to Supplementary Materials or simplifying the textual descriptions in the main manuscript.

6.While the analytical methodology for mass spectrometry is comprehensively described, certain technical details (e.g., database version, search parameters) may be moved to the Supplementary Methods/Appendix for brevity of the main text.

Reviewer #3: In Line 137, the authors state: “Here, we wanted to further investigate the role of the OBV Gc head and stalk domains, to better understand why they are apparently dispensable for multiplication in the mammalian host, yet are maintained in nature.” As the experiments were performed exclusively in mammalian cell lines rather than in a mammalian host model, the wording should be revised accordingly (e.g., replacing “mammalian host” with “mammalian cells”).

Lines 156-157. The statement that rBUNV-Δ7 exhibits “no fitness loss” in mammalian cells is too broad, as viral fitness encompasses parameters not directly assessed here. Replacing this wording with a more specific description of the measured phenotype (e.g., multiplication or virus production) would be more appropriate.

I suggest replacing “Gc-HA” with “HA-Gc” throughout the manuscript (text and figures) to accurately reflect the N-terminal position of the HA tag.

The title of Figure S4 (both in the legend and in the figure file) does not correspond to the data shown in the figure and should be corrected.

Reviewer #4: - The authors have put a lot of efforts to demonstrate the decrease association between Gc and NP (probably as RNP), but this might just be the consequence of the impaired localization of Gc, and the mechanism regarding this remains not understood. This should therefore be highlighted in the Discussion.

**********

PLOS authors have the option to publish the peer review history of their article (what does this mean?). If published, this will include your full peer review and any attached files.

If you choose “no”, your identity will remain anonymous but your review may still be made public.

**Do you want your identity to be public for this peer review?**  For information about this choice, including consent withdrawal, please see our Privacy Policy.

Reviewer #1: No

Reviewer #2: **Yes:**  Chang Li

Reviewer #3: No

Reviewer #4: No

**Figure resubmission:**

-->While revising your submission, we strongly recommend that you use PLOS’s NAAS tool (https://ngplosjournals.pagemajik.ai/artanalysis) to test your figure files. NAAS can convert your figure files to the TIFF file type and meet basic requirements (such as print size, resolution), or provide you with a report on issues that do not meet our requirements and that NAAS cannot fix.

After uploading your figures to PLOS’s NAAS tool - https://ngplosjournals.pagemajik.ai/artanalysis, NAAS will process the files provided and display the results in the "Uploaded Files" section of the page as the processing is complete. If the uploaded figures meet our requirements (or NAAS is able to fix the files to meet our requirements), the figure will be marked as "fixed" above. If NAAS is unable to fix the files, a red "failed" label will appear above. When NAAS has confirmed that the figure files meet our requirements, please download the file via the download option, and include these NAAS processed figure files when submitting your revised manuscript.

**Reproducibility:**

To enhance the reproducibility of your results, we recommend that authors of applicable studies deposit laboratory protocols in protocols.io, where a protocol can be assigned its own identifier (DOI) such that it can be cited independently in the future. Additionally, PLOS ONE offers an option to publish peer-reviewed clinical study protocols. Read more information on sharing protocols at https://plos.org/protocols?utm_medium=editorial-email&utm_source=authorletters&utm_campaign=protocols

# Author comment: Author response to Decision Letter 1

**DOI:** 10.1371/journal.ppat.1014374.r002

20 May 2026

Attachment Submitted filename: Response to reviewers 150526.docx

# Peer review: Decision Letter 1

**DOI:** 10.1371/journal.ppat.1014374.r003

**Authors:** Matthias Schnell, Alison Kell, Matthias Schnell, Alison Kell

12 Jun 2026

Dear Dr. Barr,

We are pleased to inform you that your manuscript 'The Bunyamwera orthobunyavirus Gc glycoprotein head and stalk drives an infectious virion assembly pathway specific for the insect host' has been provisionally accepted for publication in PLOS Pathogens.

Before your manuscript can be formally accepted you will need to complete some formatting changes, which you will receive in a follow up email. A member of our team will be in touch with a set of requests.

Please note that your manuscript will not be scheduled for publication until you have made the required changes, so a swift response is appreciated.

IMPORTANT: The editorial review process is now complete. PLOS will only permit corrections to spelling, formatting or significant scientific errors from this point onwards. Requests for major changes, or any which affect the scientific understanding of your work, will cause delays to the publication date of your manuscript.

Should you, your institution's press office or the journal office choose to press release your paper, you will automatically be opted out of early publication. We ask that you notify us now if you or your institution is planning to press release the article. All press must be co-ordinated with PLOS.

Thank you again for supporting Open Access publishing; we are looking forward to publishing your work in PLOS Pathogens.

Best regards,

Alison M Kell

Academic Editor

PLOS Pathogens

Matthias Schnell

Section Editor

PLOS Pathogens

Sumita Bhaduri-McIntosh

Editor-in-Chief

PLOS Pathogens

orcid.org/0000-0003-2946-9497

Michael Malim

Editor-in-Chief

PLOS Pathogens

orcid.org/0000-0002-7699-2064

***********************************************************

Reviewer Comments (if any, and for reference):

Reviewer's Responses to Questions

**Part I - Summary**

Please use this section to discuss strengths/weaknesses of study, novelty/significance, general execution and scholarship.

Reviewer #1: The authors have done a thorough job addressing the many comments from the 4 reviewers. I am satisfied with these revisions.

Reviewer #2: The authors responded all the comments and made the suggested change.

Reviewer #3: The authors have satisfactorily addressed all of my previous comments. The revised manuscript includes new experimental data that strengthen the conclusions, and the text has been revised to provide a more accurate description and interpretation of the findings. I have no further major comments and believe that the manuscript is now suitable for publication in its current form.

**********

**Part II – Major Issues: Key Experiments Required for Acceptance**

Please use this section to detail the key new experiments or modifications of existing experiments that should be absolutely required to validate study conclusions.

Generally, there should be no more than 3 such required experiments or major modifications for a "Major Revision" recommendation. If more than 3 experiments are necessary to validate the study conclusions, then you are encouraged to recommend "Reject".

Reviewer #1: (No Response)

Reviewer #2: The authors responded all the comments and made the suggested change.

Reviewer #3: (No Response)

**********

**Part III – Minor Issues: Editorial and Data Presentation Modifications**

Please use this section for editorial suggestions as well as relatively minor modifications of existing data that would enhance clarity.

Reviewer #1: (No Response)

Reviewer #2: The authors responded all the comments and made the suggested change.

Reviewer #3: (No Response)

**********

PLOS authors have the option to publish the peer review history of their article (what does this mean?). If published, this will include your full peer review and any attached files.

If you choose “no”, your identity will remain anonymous but your review may still be made public.

**Do you want your identity to be public for this peer review?**  For information about this choice, including consent withdrawal, please see our Privacy Policy.

Reviewer #1: No

Reviewer #2: **Yes:**  Chang Li

Reviewer #3: No

# Editor report: Acceptance letter

**DOI:** 10.1371/journal.ppat.1014374.r004

**Authors:** Matthias Schnell, Alison Kell, Matthias Schnell, Alison Kell

Dear Dr. Barr,

We are delighted to inform you that your manuscript, "

The Bunyamwera orthobunyavirus Gc glycoprotein head and stalk drives an infectious virion assembly pathway specific for the insect host," has been formally accepted for publication in PLOS Pathogens.

We have now passed your article onto the PLOS Production Department who will complete the rest of the pre-publication process. All authors will receive a confirmation email upon publication.

The corresponding author will soon be receiving a typeset proof for review, to ensure errors have not been introduced during production. Please review the PDF proof of your manuscript carefully, as this is the last chance to correct any scientific or type-setting errors. Please note that major changes, or those which affect the scientific understanding of the work, will likely cause delays to the publication date of your manuscript. Note: Proofs for Front Matter articles (Pearls, Reviews, Opinions, etc...) are generated on a different schedule and may not be made available as quickly.

Soon after your final files are uploaded, the early version of your manuscript, if you opted to have an early version of your article, will be published online. The date of the early version will be your article's publication date. The final article will be published to the same URL, and all versions of the paper will be accessible to readers.

For Research Articles, you will receive an invoice from PLOS for your publication fee after your manuscript has reached the completed accept phase. If you receive an email requesting payment before acceptance or for any other service, this may be a phishing scheme. Learn how to identify phishing emails and protect your accounts at https://explore.plos.org/phishing.

Thank you again for supporting open-access publishing; we are looking forward to publishing your work in PLOS Pathogens.

Best regards,

Sumita Bhaduri-McIntosh

Editor-in-Chief

PLOS Pathogens

orcid.org/0000-0003-2946-9497

Michael Malim

Editor-in-Chief

PLOS Pathogens

orcid.org/0000-0002-7699-2064
